# Spatio-temporal evolution and regional heterogeneity in the efficiency of agricultural non-point source pollution control within the Chaohu Lake Basin

**DOI:** 10.1038/s41598-026-45974-4

**Published:** 2026-03-31

**Authors:** Qingyang He, Qinzhang Han, Weixue Lu

**Affiliations:** School of Artificial Intelligence, Anhui Agricultural University, Hefei, 230036 China

**Keywords:** Agricultural non-point source pollution, Chaohu Lake Basin, SBM-DDF-GML model, Regional heterogeneity, Governance efficiency, Ecology, Ecology, Environmental sciences, Environmental social sciences, Hydrology

## Abstract

As a key grain-producing base and ecological barrier in China, the Chaohu Lake Basin plays a crucial role in ensuring food security and promoting high-quality ecological development. Uncovering the spatio-temporal evolution patterns and regional heterogeneity of agricultural non-point source pollution control efficiency within this basin is therefore crucial. Using panel data from 17 counties (districts) in the Chaohu Lake basin covering 2016–2023, this study adopts the SBM-DDF model to statically measure agricultural non-point source pollution control efficiency and applies the Global Malmquist-Luenberger (GML) index to dynamically decompose such efficiency. The findings indicate that: (1) Agricultural inputs in the Chaohu Lake basin are generally excessive, with the over-application of fertilizers and pesticides as the core issue, while the utilization efficiency of labor, machinery, and irrigation remains low. (2) Governance efficiency across entire river basins exhibits cyclical fluctuations characterised by ‘policy effectiveness—efficiency decline—adjustment recovery’. Furthermore, the findings reveal a weak synergy between technological advancement and management efficiency, which severely constrains overall governance effectiveness. (3) Significant regional heterogeneity exists: upstream areas exhibit higher excess inputs of labor, machinery, and pesticides, coupled with dual deficiencies in technology and management. Midstream regions demonstrate efficient land use and the lowest reliance on chemical pesticides, yet suffer from unstable policy implementation. Downstream areas are characterised by pronounced excesses in fertiliser and irrigation, alongside structural imbalances in technology and management. Based on these findings, this paper proposes targeted strategic recommendations to advance the green transformation of agriculture in the Chaohu Lake basin and achieve efficient control of non-point source pollution.

**Supplementary Information:**

The online version contains supplementary material available at 10.1038/s41598-026-45974-4.

## Introduction

With China’s rapid economic development and accelerated agricultural modernisation, the nation’s agricultural production system has been continuously refined. Agricultural mechanisation levels have significantly increased, while the contribution rate of scientific and technological progress has steadily grown. Grain output has remained stable above 6500 million tons for consecutive years, markedly enhancing the comprehensive agricultural production capacity. However, in the course of this development, the issue of agricultural non-point source pollution has become increasingly prominent, emerging as a key factor constraining both ecological and environmental quality and the sustainable development of agriculture. Agricultural pollutants primarily enter water bodies via surface runoff and farmland seepage, causing eutrophication in lakes and rivers. This not only disrupts the balance of aquatic ecosystems and threatens the safety and health of residents’ drinking water, but also severely constrains the green transformation of agriculture and the sustainable development of river basins^1,2^.

As one of China’s five major freshwater lakes, Chaohu Lake serves not only as a vital ecological barrier for the Jianghuai region but also as a crucial water source for agricultural production and domestic use within its watershed. In recent years, governments at all levels have stepped up governance efforts, including revising the Regulations on Water Pollution Prevention and Control in the Chaohu Lake Basin and launching demonstration projects for the comprehensive control of agricultural non-point source pollution. Although the water quality of Chaohu Lake has long been maintained at Grade IV or above, its eutrophication trend has not been fundamentally reversed^3^. The large annual application of chemical fertilizers has further aggravated eutrophication in the lake. Therefore, elucidating the evolution patterns and regional heterogeneity of agricultural non-point source pollution control efficiency in the Chaohu Lake basin is of critical importance for precisely enhancing management effectiveness and fundamentally improving the basin’s ecological environment.

Currently, while existing literature has investigated the assessment of agricultural non-point source pollution, most studies have been conducted at the national or provincial scale^2,4,5^, with relatively insufficient research focusing specifically on the Chaohu Lake basin. Meanwhile, studies related to the Chaohu Lake basin are mostly limited to localized regions or single issues^3,5–7^, lacking systematic and comprehensive integrated analyses at the full-basin scale. Consequently, it is difficult to reveal the spatiotemporal differentiation and transmission mechanisms of pollution across the entire basin. Based on this, this paper takes the Chaohu Lake basin as its research subject. By integrating the SBM-DDF and GML index models, it conducts static efficiency measurement and dynamic decomposition on panel data from 17 counties (districts) spanning 2016–2023, alongside regional heterogeneity analysis. This systematically reveals the spatiotemporal evolution patterns and regional heterogeneity characteristics of its governance efficiency.

This paper makes three principal contributions. Firstly, methodologically, the integration of SBM-DDF with the GML index enables a systematic analysis of agricultural non-point source pollution in the Chaohu Lake basin, encompassing ‘static efficiency measurement, dynamic decomposition, and regional comparison’. Secondly, through quantifying the coordination between technology and management, specific forms of imbalance such as ‘technology silos’ and ‘management compensation’ were identified. Thirdly, from a perspective based on the division of watercourses into upper, middle and lower reaches according to their flow direction, the regional heterogeneity was examined.

The remainder of this paper is structured as follows. Section “Literature review” reviews the relevant literature on agricultural non-point source pollution; Section “Research methods” introduces the methodology of the empirical model; Section “Research design” details the data sources and research design; Section “Empirical research” presents the empirical findings; Section “Conclusions and recommendations” offers conclusions and recommendations; Section “Limitations and future prospects” presents the limitations and future prospects.

## Literature review

To investigate the evolution mechanisms and regional heterogeneity of agricultural non-point source pollution control efficiency in the Chaohu Lake basin, this paper systematically reviews the origins, evaluation methods, and research progress of agricultural non-point source pollution, as well as the development of the SBM-DDF model.

### The origins of agricultural non-point source pollution

The origins of agricultural non-point source pollution are highly correlated with the transformation of agricultural production patterns and the global and regional stages of agricultural development, and can be broadly categorised into international and domestic dimensions. At the international level, the fundamental shift in agricultural production models constitutes the core driver of agricultural non-point source pollution. Globally, agriculture has transitioned from traditional ecological cycles to ‘petroleum-based agriculture’. The large-scale application of chemical inputs such as fertilizers and pesticides, while substantially increasing crop yields, has disrupted the material balance within agricultural ecosystems. This has led to a dramatic surge in pollutant emissions. By the mid-twentieth century, developed nations in Europe and America were the first to exhibit problems such as water eutrophication, notably in the Great Lakes basin of the United States and the Rhine River basin in Europe^8^. In early international regulatory practices, the United States ‘Clean Water Act’ first explicitly defined responsibilities for controlling agricultural non-point source pollution at the national legislative level in 1972. This became the world’s first targeted legislative document, marking the transition of agricultural non-point source pollution from an ‘implicit issue’ to a phase of ‘institutionalised regulation’. It provided a paradigm reference for subsequent governance efforts by other nations^6^. From a domestic perspective, the issue of agricultural non-point source pollution in China has gradually come to the fore during the process of agricultural intensification. Following 1978, China witnessed a marked annual increase in chemical fertilizer application rates. Concurrently, the scale of livestock and poultry farming continued to expand. The excessive use of chemical fertilizers, imbalances in agricultural production structures, and shifts in land use patterns have become the core mechanisms driving pollution formation^9^. During this period, the problem of eutrophication in river basins such as Chaohu Lake and Taihu Lake intensified, becoming a core challenge in controlling water pollution within these basins^7^.

### Assessment methods for agricultural non-point source pollution

In recent years, methodologies for assessing agricultural non-point source pollution have continually evolved, broadly categorised into three approaches: fundamental measurement techniques, model-based analytical methods, and integrated technological approaches. These methodologies progressively enhance both assessment accuracy and application depth, providing crucial support for pollution source tracing, load quantification, and targeted identification of remediation measures. For the first category of methods, serving as an early assessment tool, they leverage the advantages of readily available data and straightforward operation to identify the total volume and sources of pollution at a macro level. For instance, Wang et al.^6^ clarified the discharge characteristics of different pollution sources by monitoring concentrations in rural domestic sewage and livestock manure. Moreover, Zhou and Gao^3^ employed the output coefficient method to demonstrate that agricultural non-point sources contribute over 60% to nitrogen and phosphorus pollution in river basins. Sun et al.^7^ utilised material flow analysis to assess the risk of nitrogen surplus resulting from fertilizer application at the provincial scale. However, such approaches predominantly focus on the unidirectional relationship between resource inputs and pollution emissions, failing to clarify the systemic coupling mechanism among input, output, and pollution. Moreover, they are ineffective in quantifying issues related to efficiency redundancy.

To overcome these limitations, some research has shifted towards a second approach that bridges the gap between empirical statistics and process mechanisms. Among these, efficiency assessment models represented by Data Envelopment Analysis (DEA) effectively address the shortcomings of traditional methods in quantifying efficiency. For instance, Qiu and Li^5^ developed a comprehensive ‘input–output-pollution’ indicator system based on Tone’s^10^ non-radial, non-angular SBM model to assess provincial agricultural ecological efficiency. Their findings revealed that eastern regions exhibited significantly higher efficiency values than central and western regions due to technological advantages.

For the third category of methods, this approach integrates multiple technical means, enabling effective characterization of the dynamic evolution of pollution and identification of spatial heterogeneity. For instance, Zhu et al.^11^ developed the ITO3dE model, integrating GIS spatial matrices, kernel density analysis, and Getis-Ord Gi* analysis to reveal the spatio-temporal differentiation and clustering characteristics of agricultural non-point source pollution in Chongqing. In recent years, with the advancement of data science, machine learning methods (such as random forests and neural networks) have been widely applied to identify key drivers of agricultural non-point source pollution and simulate its complex nonlinear processes^12,13^. Such research deepens mechanistic understanding from a ‘process analysis’ perspective. In contrast, this paper adopts a ‘performance evaluation’ approach based on the production frontier, focusing on measuring the comprehensive effectiveness and improvement potential of management from an ‘input–output’ systems perspective. These two paradigms have distinct focuses and complement each other. This study centers on the assessment and diagnosis of governance efficiency, hence selecting the SBM-DDF-GML model system, which is more aligned with this objective.

### Development of the SBM-DDF model

The core of evaluating agricultural non-point source pollution control efficiency lies in constructing measurement tools capable of capturing multidimensional objectives encompassing ‘economic output—resource input—environmental impact’. Data Envelopment Analysis (DEA) has emerged as the mainstream method in this field due to its advantages, including the absence of prerequisites for production function forms and its ability to handle multi-input, multi-output systems. However, traditional DEA models (e.g., CCR, BCC) rely on radial and angular assumptions, requiring all inputs (or outputs) to be reduced (or expanded) proportionally. This approach fails to address non-radial slack between inputs and outputs and cannot accommodate reduction targets for undesired outputs, leading to systematic overestimation of efficiency values^10^.To overcome these limitations, Tone^10^ proposed the Slack Variable-Based Model (SBM), which incorporates input excess, desired output deficiency, and undesired output excess directly into the objective function. This enables non-radial, non-angular efficiency measurement, significantly improving the accuracy of identifying resource allocation inefficiencies. However, the SBM model only reflects the aggregate level of efficiency loss and cannot set differentiated improvement directions for inputs, expected outputs, and unintended outputs according to decision-maker preferences. To address this, Fukuyama and Weber^14^ combined the SBM model with a directional distance function (DDF) to construct the SBM-DDF model. By introducing directional vectors, this model enables decision units to optimize along specific trajectories, effectively addressing the traditional DDF model’s oversight of slack variables. It rapidly became a core tool for industrial energy conservation and emission reduction as well as agricultural environmental efficiency assessment. However, its static nature prevents exploration of efficiency changes over time, and its production frontier lacks intertemporal comparability.

The evaluation of agricultural non-point source pollution control efficiency represents an evolution from radial to non-radial, from static to dynamic, and from single efficiency measures to ‘efficiency-productivity-slack’ assessments. This paper applies the SBM-DDF model to study agricultural non-point source pollution control efficiency at the county level in the Chaohu Lake basin. This not only expands the application boundaries of this cutting-edge tool but also addresses the dual demands of basin governance for ‘management effectiveness diagnosis’ and ‘dynamic tracking evaluation’.

## Research review

In the existing literature, studies on agricultural non-point source pollution in the Chaohu Lake basin have primarily focused on pollution load calculations or localized regional analyses, exhibiting the following four shortcomings. First, the assessment perspective is limited. Existing research predominantly centers on calculating total pollution loads, with few studies establishing an efficiency analysis framework encompassing ‘inputs-expected outputs-unexpected outputs’ to identify key bottlenecks such as fertilizer labor and underutilized technologies. Second, there is a lack of systematic efficiency assessments based on basin-wide county panel data that integrate static and dynamic perspectives. Third, existing studies predominantly examine inputs or outputs at the end of the process, failing to quantify the synergistic or imbalanced mechanisms of ‘technology’ and ‘management’ in the governance process. Fourth, regional analyses often remain at the level of descriptive comparisons, unable to deeply analyze the causes of heterogeneity from the perspective of spatial transmission within the basin.

## Research methods

This paper introduces the SBM-DDF model, incorporating multiple inputs such as labor, land, and machinery, alongside expected and undesirable outputs, by constructing a production possibility set that includes undesirable outputs. Utilizing the global Malmquist-Luenberger (GML) index, it decomposes total factor productivity within the SBM-DDF model to distinguish between changes in technical efficiency and contributions from technological progress. This approach resolves intertemporal comparison issues arising from inconsistent production frontiers across different periods. Combining the SBM-DDF model with the GML index forms a three-pronged analytical framework of ‘static measurement—dynamic decomposition—causal analysis,’ establishing a cutting-edge paradigm in contemporary environmental efficiency research. Its strength lies in handling complex systems with multiple inputs and outputs (including unintended outputs), while identifying efficiency losses and improvement potential by calculating the distance between decision units and the common production frontier.

This study employs agricultural production data from 17 counties within the Chaohu Lake basin between 2016 and 2023 as its research sample. Each county constitutes an independent decision-making unit (DMU) for each year, defined by its specific input–output combination (comprising six input indicators, one expected output indicator, and two undesired output indicators). This yields a total of 136 DMUs, which serve as the evaluation sample for the model. To systematically reveal the evolution patterns of agricultural non-point source pollution control efficiency and analyse its regional heterogeneity, this paper outlines the specific steps of the research methodology as follows:

### Definition of direction vectors

To elucidate the system coupling mechanism of ‘input–output-pollution’ within the Chaohu Lake basin, the direction vector is defined as follows:

(1) When $f_{x} = 0$, the direction vector equals the actual indicator values of each DMU, reflecting ‘optimisation according to current scale proportions’. The formula is as follows:

$$\vec{g}_{x} = X^\prime ,\vec{g}_{y} = Y^\prime ,\vec{g}_{z} = Z^\prime$$

where $\vec{g}_{x} = (g_{x1} ,g_{x2} ,...,g_{x6} )$ denotes the input direction vector, $\vec{g}_{y} = (g_{y1} )$ denotes the expected output direction vector, $\vec{g}_{z} = (g_{z1} ,g_{z2} )$ denotes the undesired output direction vector; $X^\prime$ denotes the transpose of the input matrix, $Y^\prime$ denotes the transpose of the expected output matrix, $Z^\prime$ denotes the transpose of the undesired output matrix.

(2) When $f_{x} = 1$, the direction vector equals the full-sample range of each indicator. By providing all DMUs with a unified improvement benchmark independent of their own scale, this effectively eliminates the interference of scale differences, significantly enhancing the cross-regional comparability of efficiency assessment results. The formula is as follows:

$$gxj = max(Xj) - min(Xj),gyj = max(Yj) - min(Yj),gzj = max(Zj) - min(Zj)$$

### Non-radial SBM direction distance function

Traditional distance functions based on radial distance exhibit two major shortcomings in efficiency measurement: the tendency of DEA models to overestimate efficiency and an inability to handle slack. To address this, this paper adopts the methodology proposed by Fukuyama and Weber^14^, to construct the SBM-DDF objective function, formulated as follows:

1 $$min\vec{D}_{0}^{{{\mathrm{SBM}}}} {\mathrm{(X,Y,Z}};\vec{g}) = \frac{1}{2}\left[ {\frac{1}{m}\sum\limits_{i = 1}^{m} {\frac{{s_{{x_{i} }} }}{{g_{{x_{i} }} }}} + \frac{1}{s + q}\left( {\sum\limits_{r = 1}^{s} {\frac{{s_{{y_{r} }} }}{{g_{{y_{r} }} }}} + \sum\limits_{k = 1}^{q} {\frac{{s_{{z_{k} }} }}{{g_{{z_{k} }} }}} } \right)} \right]$$

where $m = 6$ denotes the number of input indicators, $s = 1$ denotes the number of expected output indicators, and $q = 2$ denotes the number of undesired output indicators; $s_{{x_{i} }}$ denotes the slack variable for the Ith input term, $s_{{y_{r} }}$ denotes the deficiency variable for the expected output of the rth item, and $s_{{z_{k} }}$ denotes the redundancy variable for the kth undesirable output; $\vec{D}_{0}^{{{\mathrm{SBM}}}}$ denotes the SBM-DDF distance value (efficiency loss), where a higher value indicates lower efficiency.

On the basis outlined above, when establishing the constraint conditions (Variable Returns to Scale, VRS), the objective function must satisfy the following constraints to ensure the rationality of the production frontier. The formula is as follows:

$$\sum\limits_{j = 1}^{136} {\lambda_{j} } X_{ij} + s_{{x_{i} }} = X_{i0}$$

$$\sum\limits_{j = 1}^{136} {\lambda_{j} } Y_{rj} - s_{{y_{i} }} = Y_{r0}$$

$$\sum\limits_{j = 1}^{136} {\lambda_{j} } Z_{kj} + s_{{z_{k} }} = Z_{i0}$$

$$\sum\limits_{j = 1}^{136} {\lambda_{j} } = 1$$

$$\lambda_{j} \ge 0,s_{{x_{i} }} \ge 0,s_{{y_{r} }} \ge 0,s_{{z_{k} }} \ge 0$$

where $X_{i0}$ denotes the initial income value; r = 1; $Y_{r0}$ denotes the initial expected output value; k = 1,2; $Z_{i0}$ denotes the initial undesirable output value; $\lambda_{j}$ represents the weight for the jth DMU.

### Construction and decomposition of the GML index

Traditional ML indices exhibit two major limitations in dynamic efficiency analysis: firstly, inter-period non-cyclability; secondly, the tendency for linear programming solutions to yield infeasible outcomes. This paper adopts the methodology proposed by Oh^15^, to define the production frontier for all samples from 2016 to 2023 and calculate the GML index. It further decomposes total factor productivity into changes in technical efficiency (EC) and changes in technical progress (TC). The specific steps are as follows:

(1) Define the production frontier surface for all samples from 2016 to 2023, using the formula:

2 $$P^{G} = U_{t = 2016}^{2023} P^{t}$$

where $P^{t}$ denotes the ‘current production possibility set’ for period t, and $P^{G}$ represents the global frontier surface.

(2) Calculate the GML index (change in total factor productivity), using the GML index to measure efficiency changes from period t to period t + 1, with the formula being:

3 $$GML_{t,t + 1} = \frac{{1 + \vec{D}_{0}^{G} (X_{t} ,Y_{t} ,Z_{t} ;\vec{g})}}{{1 + \vec{D}_{0}^{G} (X_{t + 1} ,Y_{t + 1} ,Z_{t + 1} ;\vec{g})}}$$

where $\vec{D}_{0}^{G}$ denotes the SBM-DDF distance value based on the global frontier surface $P^{G}$, $X_{t}$, ${\mathrm{Y}}_{t}$, and $Z_{t}$ represent the input, expected output, and undesirable output matrices for year t respectively. $GML_{t,t + 1} > 1$ indicates an increase in total factor productivity, whilst $GML_{t,t + 1} < 1$ signifies a decrease.

(3) Decomposing total factor productivity into changes in technical efficiency (EC) and changes in technical progress (TC), with the decomposition relationship being $GML_{t,t + 1} = EC_{t,t + 1} \times TC_{t,t + 1}$. The decomposition formula is as follows:

4 $$EC_{t,t + 1} = \frac{{1 + \vec{D}_{0}^{t} (X_{t} ,Y_{t} ,Z_{t} ;\vec{g})}}{{1 + \vec{D}_{0}^{t} (X_{t + 1} ,Y_{t + 1} ,Z_{t + 1} ;\vec{g})}}$$

where $EC > 1$ indicates management optimisation, $EC < 1$ indicates management inefficiency.

5 $$TC_{t,t + 1} = \frac{{(1 + \vec{D}_{0}^{G} (X_{t} ,Y{}_{t},Z_{t} ;\vec{g})) \times (1 + \vec{D}_{0}^{t} (X_{t + 1} ,Y_{t + 1} ,Z_{t + 1} ;\vec{g}))}}{{(1 + \vec{D}_{0}^{t} (X_{t} ,Y{}_{t},Z_{t} ;\vec{g})) \times (1 + \vec{D}_{0}^{G} (X_{t + 1} ,Y_{t + 1} ,Z_{t + 1} ;\vec{g}))}}$$

where $TC > 1$ denotes technological advancement, $TC < 1$ denotes technological backwardness.

### Calculation of relative efficiency values

To facilitate cross-unit and cross-period comparative and comprehensive analysis of the Chaohu Lake basin, the calculated distance function values were converted into a directly comparable relative efficiency index ranging from 0 to 1.

1. When $f_{x} = 0$, the calculation formula is $eff = \frac{1}{{1 + \vec{D}_{0}^{SBM} }}$.
2. When $f_{x} = 1$, the calculation formula is $eff = 1 - \vec{D}_{0}^{{{\mathrm{SBM}}}}$.

### Model applicability and robustness

Prior to applying the SBM-DDF model, this paper conducted systematic model setup and validation work to ensure the rationality of model construction. It also compared results derived from different direction vectors to guarantee the robustness of conclusions.

First, considering the significant variations in agricultural scale, topography, and resource endowments across counties (districts) within the Chaohu Lake basin, agricultural production likely deviates from the constant returns to scale (CRS) assumption. Therefore, drawing on the methodology of Khan et al.^16^, this study adopted the variable returns to scale (VRS) assumption when constructing the SBM-DDF model. This setting allows decision units to achieve optimal efficiency at different scales, enabling more accurate separation of ‘pure technical efficiency’ determined by pure technology and management levels. This prevents misclassifying ‘scale inefficiency’ caused by excessively large or small operations as ‘management inefficiency,’ making efficiency assessment results more realistic. Second, the management efficiency of agricultural non-point source pollution in the Chaohu Lake basin examined in this study measures the relative capacity of 17 counties (districts) to maximize agricultural economic output while minimizing resource inputs and negative environmental impacts under given conditions.

The static efficiency and dynamic decomposition results in this paper are both derived based on the assumption that the direction vector is 0 (i.e., optimization is conducted according to the input–output scale ratio of the decision-making unit itself). This assumption aligns with the logic of incremental improvements based on existing production structures in management practices across counties and districts. To test the sensitivity of the conclusions to directional settings, this paper simultaneously calculates the results with a direction vector of 1 (i.e., using the full sample range as a unified improvement) and conducts paired sample t-tests. The comparison results for slack values are presented in Appendix Table 1, while the comparison results for GML indices are shown in Appendix Table 2.

**Table 1:** Indicator system.

Indicator type	Indicator name	Indicator symbol	Indicator meaning
Input indicators	Labor input	X1	Number of persons employed in agriculture, forestry, animal husbandry and fishery
	Land input	X2	Total area under crops
	Machinery input	X3	Total power of agricultural machinery
	Irrigation input	X4	Effective irrigated area
	Pesticide input	X5	Pesticide application rate
	Fertilizer input	X6	Application rate of chemical fertilizers
Expected output indicators	agricultural output value	Y1	Agricultural output value at constant 2012 prices
Undesirable output indicators	Agricultural carbon emissions	Z1	Total carbon emissions from fertilizers, pesticides, total mechanical power, irrigated area, and crop cultivation area
	Agricultural non-point source pollution	Z2	Total emissions of nitrogen and phosphorus

**Table 2:** Carbon emission factors.

Carbon emissions targets	Carbon emission factor	Unit	Reference source
Reference source	0.8956	Kg/Kg	West and Marland^20^
Crop area under cultivation	16.47	t/khm^2^	West and Marland^20^
Total Agricultural Machinery Power	0.018	t/10,000 kW	West and Marland^20^
Effective irrigated area	20.476	Kg/hm^2^	HU et al.^17^
Pesticide application rate	4.93	Kg/kg	Hussain et al.^8^

As can be seen from Appendix Tables 1 and 2, the slack variables and GML indices for the two sets of results are highly consistent. Results under both settings reveal that the correlation coefficients between the slack values of the 9 inputs and 3 outputs range from 0.977 to 1.000 (*p* < 0.001). This indicates that regardless of whether the model tends toward radial minimization improvements or directional improvements, the identified resource misallocations (e.g., the slack in machinery inputs was 0.1206 in 2017 and decreased to 0.0610 in 2023, indicating improved misallocation of mechanical resources, as shown in Table 3) and environmental pollution issues (e.g., excessive fertilizer and pesticide inputs lead to excessive total nitrogen and phosphorus emissions, causing environmental pollution in Chaohu Lake) are effectively captured. decreasing to 0.0610 in 2023, indicating improved machinery resource allocation) and environmental pollution issues (e.g., excess fertilizer and pesticide inputs leading to excessive total nitrogen and phosphorus emissions, causing eutrophication in the Chaohu Lake basin) are fundamentally consistent in structure and magnitude. Additionally, the correlation coefficients for total factor productivity (GML), technological change (TC), and technological efficiency change (EC) under the two settings were 0.905, 0.962, and 0.959, respectively. This confirms the high robustness of the core conclusions regarding the spatiotemporal evolution trends and driving factors of governance efficiency in the Chaohu Lake basin, which remain unaffected by the selection of technical parameters such as direction vectors.

**Table 3:** Annual investment slack mean.

Year	Labor input	Land input	Machinery input	Irrigation input	Pesticide input	Fertilizer input
2016	0.1445	0.1585	0.1193	0.2315	0.1516	0.1505
2017	0.1505	0.1702	0.1206	0.2399	0.1802	0.2031
2018	0.1248	0.0706	0.0878	0.1218	0.1709	0.1712
2019	0.1463	0.0686	0.0880	0.1263	0.1620	0.1578
2020	0.1183	0.0646	0.0832	0.1164	0.1325	0.1227
2021	0.1165	0.0639	0.0789	0.1153	0.1222	0.1301
2022	0.1210	0.0715	0.0801	0.1283	0.1264	0.1593
2023	0.1144	0.0517	0.0610	0.0677	0.1076	0.1135

## Research design

### Development of the indicator system

Given the dynamic nature of agricultural non-point source pollution and data availability, this study covers the period from 2016 to 2023. In selecting the sample areas, consideration was given to the actual distribution of agricultural production within the Chaohu Lake basin and the scope of non-point source pollution impacts. Consequently, the following locations were ultimately chosen: Yaohai District, Luyang District, Shushan District, Baohe District in Hefei City; Chaohu City (county-level); Feixi County, Feidong County, Lujiang County, Changfeng County; Jiujiang District of Wuhu City and Wuwei City at county level; Hanshan County and He County of Ma’anshan City; Jinan District, Shucheng County, and Huoshan County of Liu’an City; and Yuexi County of Anqing City(The abbreviations used for administrative area names in this document are listed in Appendix 3.). Simultaneously, the 17 counties (districts) are categorised into three zones according to water flow direction: upper, middle, and lower reaches. The upper reaches comprise Yuexi County, Huoshan County, Shucheng County, Jinan District, and Changfeng County; the middle reaches include Yaohai District, Luyang District, Shushan District, Baohe District, Feixi County, Feidong County, Lujiang County, and the county-level city of Chaohu; The downstream region comprises Hanshan County, He County, the county-level city of Wuwei, and Jiujiang District.

In order to systematically analyse the mechanisms underlying the evolution of agricultural non-point source pollution control efficiency and the regional variations within the Chaohu Lake basin, this paper draws on indicators selected from a number of authoritative sources to establish a suitable indicator system^2,8,17^. This system encompasses three categories of variables: inputs, expected outputs, and undesired outputs, See Table 1 for details. The inclusion of agricultural carbon emissions (Z1) in the evaluation system for non-point source pollution control efficiency is primarily justified by two key reasons. First, carbon emissions stem predominantly from the use of inputs such as chemical fertilizers, pesticides, and diesel fuel, whose driving sources align closely with nitrogen and phosphorus pollution. Second, based on China’s ‘dual carbon’ strategy and the requirement for coordinated advancement of ‘pollution reduction and carbon reduction’ in agricultural green development^17^, this indicator comprehensively reflects the environmental pressure exerted by agricultural activities, thereby enhancing the systematic nature of efficiency assessments. At the same time, empirical research has confirmed that anthropogenic inputs of carbon, nitrogen and phosphorus are intrinsically interlinked; agricultural production is both a source of greenhouse gas emissions and a common source of nitrogen and phosphorus leaching^18^. This interlinkage provides a basis for incorporating agricultural carbon emissions—as an unintended by-product—into evaluation frameworks alongside indicators of nitrogen and phosphorus pollution, thereby facilitating a more comprehensive assessment of the overall environmental pressures arising from agriculture.

## Research data

The sample data in this paper are sourced from the Anhui Statistical Yearbook, the Anhui Rural Statistical Yearbook, and local statistical yearbooks. For missing values, this study employs the linear interpolation method adapted from Lai et al.^19^. Given the challenges in obtaining annual-scale, actual monitored carbon emission data for each county within the basin, this study adopts the widely recognized ‘emission factor method’ from existing research^17^ to estimate agricultural carbon emissions. To derive specific agricultural carbon emission values for the Chaohu Lake basin, this study selected five indicators—fertilizer, pesticides, total mechanical power, effective irrigated area, and crop planting area—to estimate agricultural carbon emissions. Each indicator was multiplied by its corresponding emission coefficient, as detailed in Table 2.

At the same time, due to issues such as excessive range in the raw data and significant differences in the magnitude of indicator values, all data underwent normalization processing to eliminate dimensional effects and meet model requirements. The formula is as follows:

$$Z = 0.1 + 0.9 * \frac{X - \min \left( X \right)}{{\max \left( X \right) - \min \left( X \right)}}$$

where, *Z* denotes the normalised data; *X* denotes the raw data; $\min \left( X \right)$ and $\max \left( X \right)$ denote the minimum and maximum values of the indicator data respectively. Since this paper focuses on relative efficiency comparisons, this treatment does not affect the direction of the conclusions.

## Empirical research

This paper systematically reveals the scope for optimising inputs and outputs in agricultural non-point source pollution control within the Chaohu Lake basin, the patterns of dynamic efficiency evolution, and regionally differentiated characteristics. Based on the results from the SBM-DDF-GML model, it conducts empirical analysis across three dimensions: slack variables, technology and management, and regional heterogeneity.

### Input–output slack analysis

To better investigate the trends in slack variables within the Chaohu Lake basin, this study analyses data from both annual and county-level dimensions.

#### Slack variable timing analysis

**Fig. 1:**
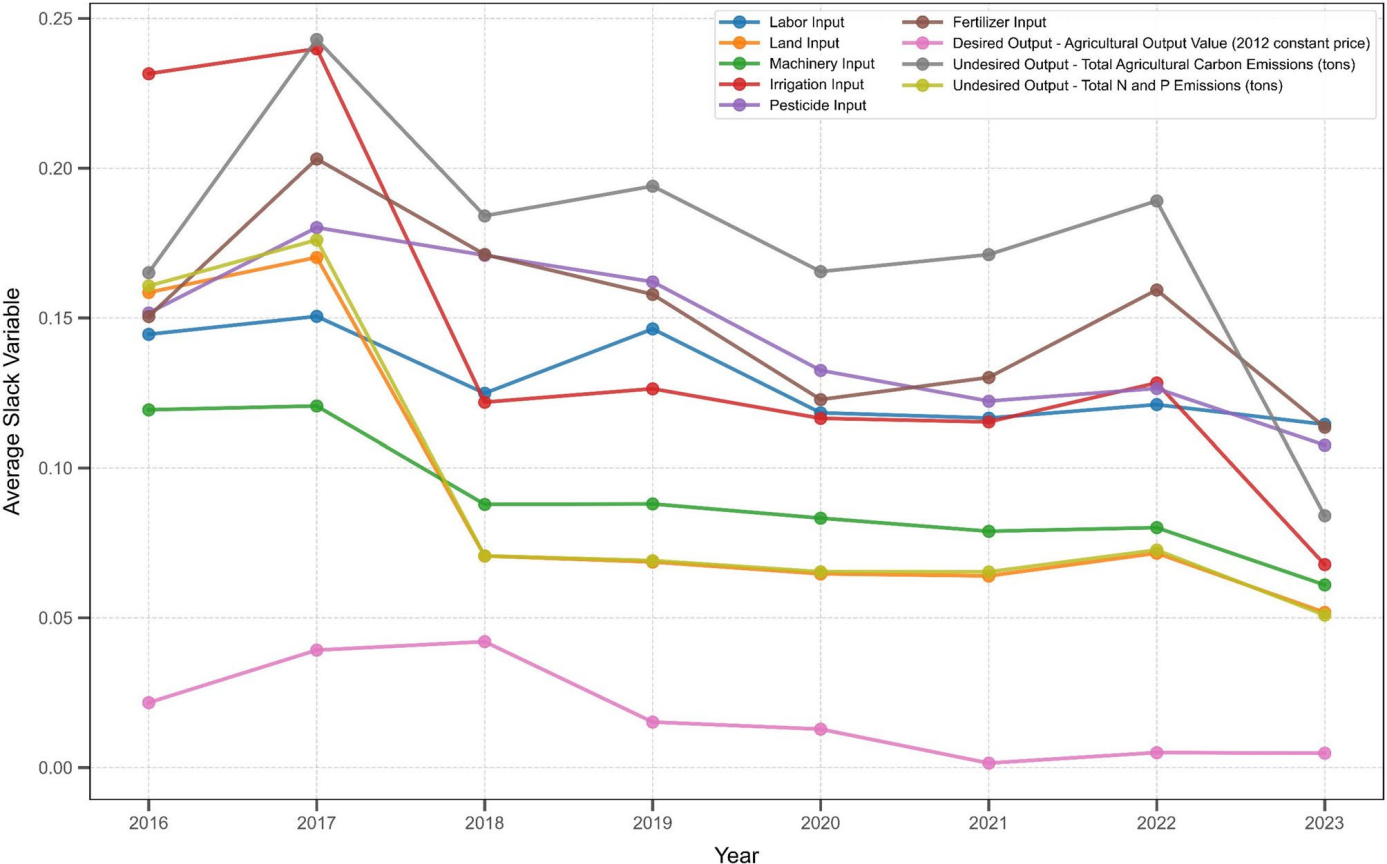
Annual change in slack variables for each indicator.

This study employs the SBM-DDF-GML model to calculate the input and output slack values for each decision-making unit (DMU). Through numerical computation, the annual mean slack values for input and output indicators across the Chaohu Lake basin were determined. The mean input slack values are detailed in Table 3, while the mean output slack values are presented in Table 4. These data were plotted as line charts, as shown in Fig. 1.

**Table 4:** Annual output slack mean.

Year	Expected output	Undesirable output—Agricultural carbon emissions	Undesirable output—Total emissions of nitrogen and phosphorus
2016	0.0216	0.1651	0.1607
2017	0.0392	0.2429	0.1760
2018	0.0420	0.1841	0.0706
2019	0.0152	0.1940	0.0691
2020	0.0128	0.1655	0.0653
2021	0.0014	0.1711	0.0653
2022	0.0050	0.1890	0.0726
2023	0.0047	0.0841	0.0509

As shown in Fig. 1, the slack values for all inputs and unintended outputs decreased significantly in tandem during 2017–2018, providing direct evidence of the immediate effectiveness of the 2017 ‘Beautiful Chaohu’ policy series. Notably, the slack curves for chemical fertilizers and pesticides remained consistently elevated, highlighting them as the primary sources of labor throughout the study period.

As shown in Fig. 1, the simultaneous decline in input slack and undesired output slack during 2017–2018 may be closely linked to the introduction and implementation of policies such as the 2017 ‘Work Plan on Accelerating the Development of a Beautiful and Green Chaohu Lake’ and the 2017 ‘Opinions on Building a Beautiful and Green Chaohu Lake’^21,22^. Therefore, 2018 marks a pivotal juncture for enhancing the efficiency of governance within the Chaohu Lake basin. Consequently, 2018 also marked a pivotal juncture for enhancing the efficiency of governance within the Chaohu Lake basin. Furthermore, the slack values for fertilizers and pesticides remained persistently high throughout the 2016–2023 period, indicating that these two elements constitute the primary surplus in agricultural inputs and represent key factors influencing the management of non-point source pollution within the Chaohu Lake basin.

As shown in Table 3, labor input slack peaked in 2017 before fluctuating and declining to 0.1144 by 2023. This reflects a potential trend towards more rational labor allocation, or a sustained shift of agricultural labor leading to a decline in the workforce. Concurrently, rising agricultural labor costs are compelling operators to plan their workforce more meticulously. Land input intensity peaked in 2017 before declining to 0.0517 by 2023. This indicates improved efficiency in utilising cultivated areas, linked to the adoption of large-scale farming practices such as family farms and cooperatives. These models enable contiguous planting, reduce land wastage from features like field ridges and drainage ditches, and support the development of high-standard farmland. The machinery input slack coefficient stood at 0.1206 in 2017, declining to 0.0610 by 2023. This indicates an improvement in the misallocation of mechanical resources, potentially attributable to cooperative machinery sharing, cross-regional machinery operations, and advancements in resource allocation techniques. Irrigation input slack peaked in 2016 and decreased to 0.0677 by 2023, demonstrating the remarkable effectiveness of water-saving irrigation projects. The pesticide input intensity reached 0.1802 in 2017, declining to 0.1076 by 2023. Meanwhile, the fertilizer input intensity fluctuated from 0.2031 in 2017 to 0.1135 in 2023. This reflects the effectiveness of policies promoting reduced fertilizer and pesticide use, including organic fertilizer substitution for chemical fertilizers and green pest control measures.

As shown in Table 4, the expected output slack peaked in 2018, declining to 0.0014 by 2021, indicating a significant improvement in efficiency. However, a slight increase in slack was observed in 2023, suggesting that certain regions may have insufficient industrial chain extension, thereby limiting the potential for value-added enhancement. The mean value of non-intended agricultural carbon emissions reached its peak in 2017 and declined by 2023, indicating the effectiveness of clean energy substitution and standardised agricultural machinery operations. The mean slack values for total nitrogen and total phosphorus emissions peaked in 2017, declining to 0.0509 by 2023. Analysis of Table 3 indicates that reduced redundancy in inputs such as pesticides, fertilizers, and machinery directly led to decreased carbon emissions alongside nitrogen and phosphorus discharges. Concurrently, the construction of constructed wetlands and ecological ditches within the Chaohu Lake basin further enhanced pollutant retention and purification.

#### Slack variable county-level analysis

**Fig. 2:**
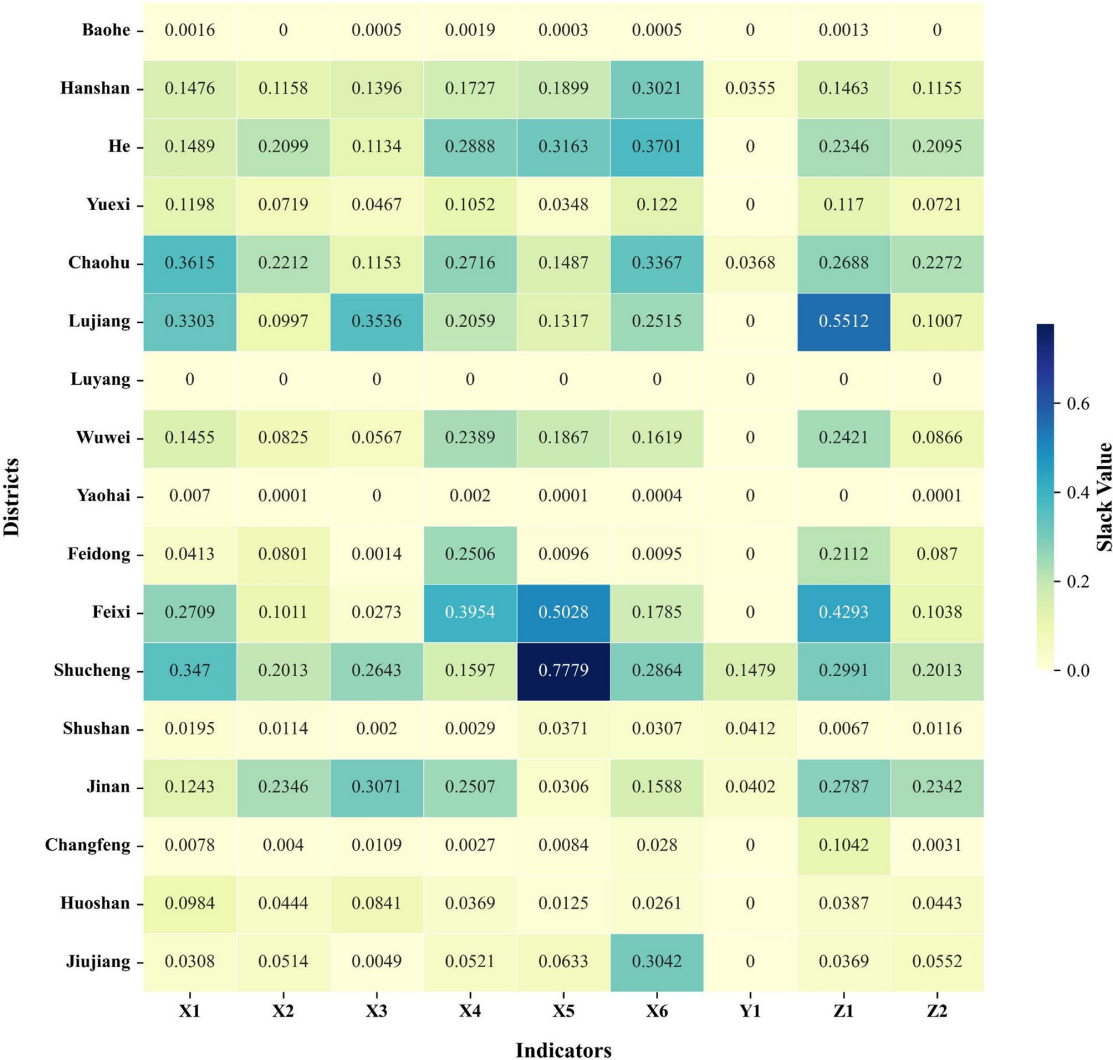
Average values of Slack variables across counties in the Chaohu Lake basin. The ring-shaped high-pollution belt around Chaohu Lake.

Based on the specific results of the slack variables for each county-level indicator, this paper has produced a heatmap illustrating the mean values of slack variables across counties within the Chaohu Lake basin. This provides a visual representation of the disparities between county-level areas, as detailed in Fig. 2.

Figure 2 clearly reveals the disparate patterns of pollution across counties. The high-pollution group (such as Lujiang County and He County) is concentrated around the main body of Chaohu Lake and downstream of its major tributaries, forming a ring-shaped pollution belt around the lake. In contrast, the low-pollution group is predominantly located in mountainous areas at the periphery of the watershed or in main urban centers, exhibiting distinct spatial clustering characteristics. As shown by the results in Fig. 2, the degree of non-point source pollution in the Chaohu Lake basin can be categorised into three levels: low, medium, and high.

For the low-pollution group (comprising Baohe District, Luyang District, Yaohai District, Shushan District, Changfeng County in Hefei City, Yuexi County in Anqing City, and Huoshan County in Liu’an City), the primary characteristics are core pollution indicators Z1 ≤ 0.12 and Z2 ≤ 0.08, representing the lowest pollution levels across the entire basin. Based on their internal differences and shared characteristics, the low-pollution group can be further subdivided into a non-agricultural activities subgroup (Baohe District, Luyang District, Yaohai District) and an ecological agriculture subgroup (Yuexi County, Huoshan County, Shushan District, Changfeng County).

For the subgroup with no agricultural activity, this area constitutes the urban core, characterised by the absence of large-scale farming. Only Yaohai District exhibits scattered agricultural practices, with minimal inputs of labor, machinery, and land, resulting in virtually zero agricultural pollution levels. For the ecological agriculture subgroup, Z1 and Z2 values in this area are relatively low across the entire watershed. Yuexi County and Huoshan County focus primarily on tea and medicinal herb cultivation, employing precise inputs with minimal fertilizer and pesticide use, resulting in no surplus pollution. Shushan District has only limited ecological agriculture, with extremely low pollution levels. Changfeng County features large-scale strawberry cultivation, exhibiting a slight surplus of chemical fertilizers.

For the moderately polluted group (comprising Feidong County in Hefei, Jiujiang District in Wuhu, Wuwei City, and Hanshan County in Ma’anshan), the core pollution characteristic is ‘low input efficiency resulting in controllable pollution, yet optimisation is required’. While no extreme pollution exists, targeted emission controls are necessary. For instance, Feidong County exhibits slightly higher irrigation input redundancy due to ageing irrigation infrastructure; Jiujiang District exhibits redundant fertilizer inputs in vegetable cultivation (X6 = 0.3042), though pollution remains manageable within input scales; Wuwei City demonstrates relatively high irrigation input redundancy alongside minor pesticide and fertilizer surpluses; Hanshan County, a primary rice-producing area, shows significant fertilizer redundancy exacerbated by soil degradation, creating a vicious cycle.

For the high-pollution group (comprising Lujiang County, Feixi County, and Chaohu City in Hefei; He County in Ma’anshan; and Shucheng County and Jinan District in Lu’an), the core issue is ‘excessive inputs leading to uncontrolled pollution’. This group exhibits the highest pollution intensity across the entire basin and possesses clearly identifiable ‘high-pollution sources’, making it a key area for addressing non-point source pollution in Chaohu Lake. Based on their internal variations and shared characteristics, these areas are subdivided into a high carbon emissions subgroup (Lujiang County, Feixi County) and a high nitrogen-phosphorus pollution subgroup (He County, Shucheng County, Jinan District, Chaohu City).

For the high-carbon-emission subgroup, Lujiang County’s Z1 basin recorded the highest pollution levels. Mechanical redundancy led to inefficient operations, compounded by pesticide and fertilizer wastage causing uncontrolled carbon emissions. In Feixi County, redundant irrigation and pesticide inputs, coupled with frequent agricultural machinery use, resulted in elevated carbon emissions. For the high nitrogen-phosphorus pollution subgroup, He County’s Z2 sub-basin recorded elevated levels. Extensive vegetable cultivation resulted in excessively high fertilizer application per mu, with runoff carrying nitrogen and phosphorus into the lake. Jinan District exhibited redundant machinery, irrigation, and land inputs coupled with insufficient output. Chaohu City exhibits redundant chemical fertilizer and labor inputs. Its proximity to the main body of Chaohu Lake places it at greatest risk from nitrogen and phosphorus pollution. Shucheng County demonstrates extreme pesticide redundancy coupled with insufficient output. Its high proportion of hilly terrain means tea harvesting relies heavily on manual labor. As young and middle-aged workers migrate for employment, the ageing of the remaining labor force has reduced harvesting efficiency, resulting in labor redundancy.

### Analysis of technology-management coordination

To systematically investigate the synergistic relationship between agricultural technological progress and management efficiency in the Chaohu Lake basin, this study analyses the phenomenon across five dimensions: sample typology classification, fluctuations in overall governance efficiency, structural imbalances, coupling coordination levels, and county-level performance.

#### Classification of sample types

This study calculates the degree of inefficiency in governance effectiveness (INEFF = 1—EFF) within the Chaohu Lake basin. The average INEFF values for the high-efficiency and medium-efficiency zones were found to be 0.1225 and 0.3268 respectively, corresponding to resource wastage rates of 12.25% and 32.68%. This indicates significant hierarchical differences in governance efficiency, as detailed in Table 5.

**Table 5:** Efficiency grade distribution.

Efficiency rating	Sample size	INEFF mean	Resource wastage rate
High-efficiency zone	103	0.1225	12.25%
Medium-efficiency zone	33	0.3268	32.68%

**Fig. 3:**
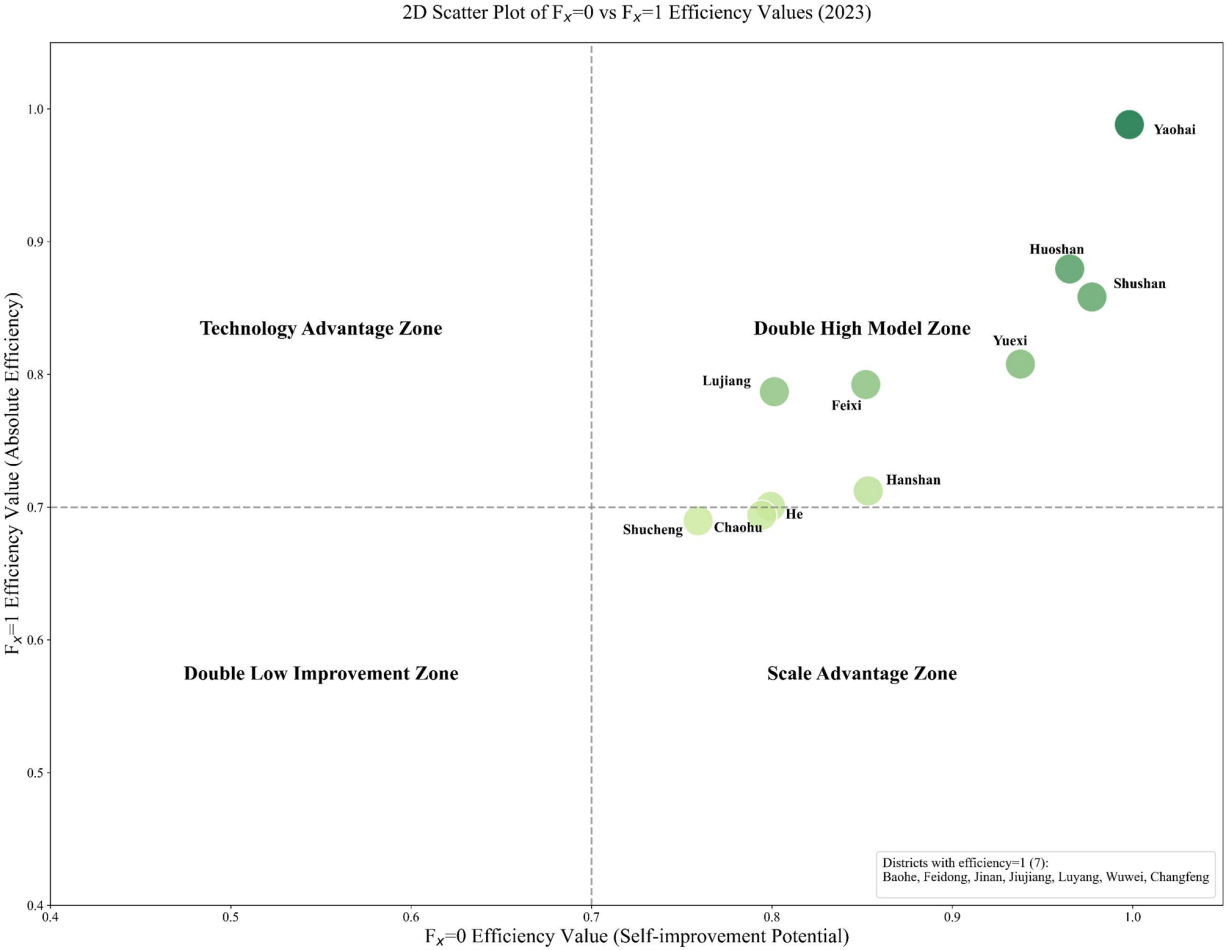
Two-dimensional scatter plot of efficiency values.

**Fig. 4:**
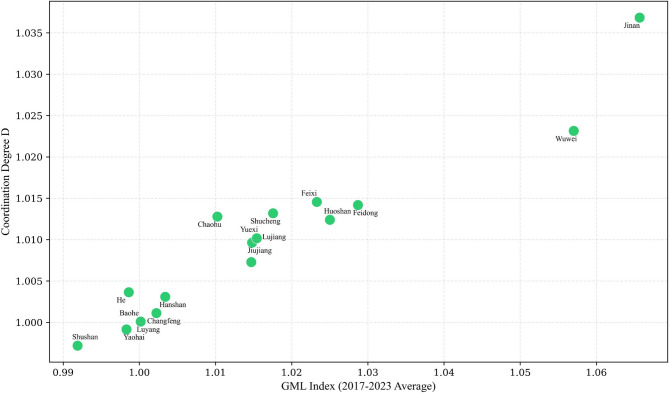
GML index and coordination degree.

**Fig. 5:**
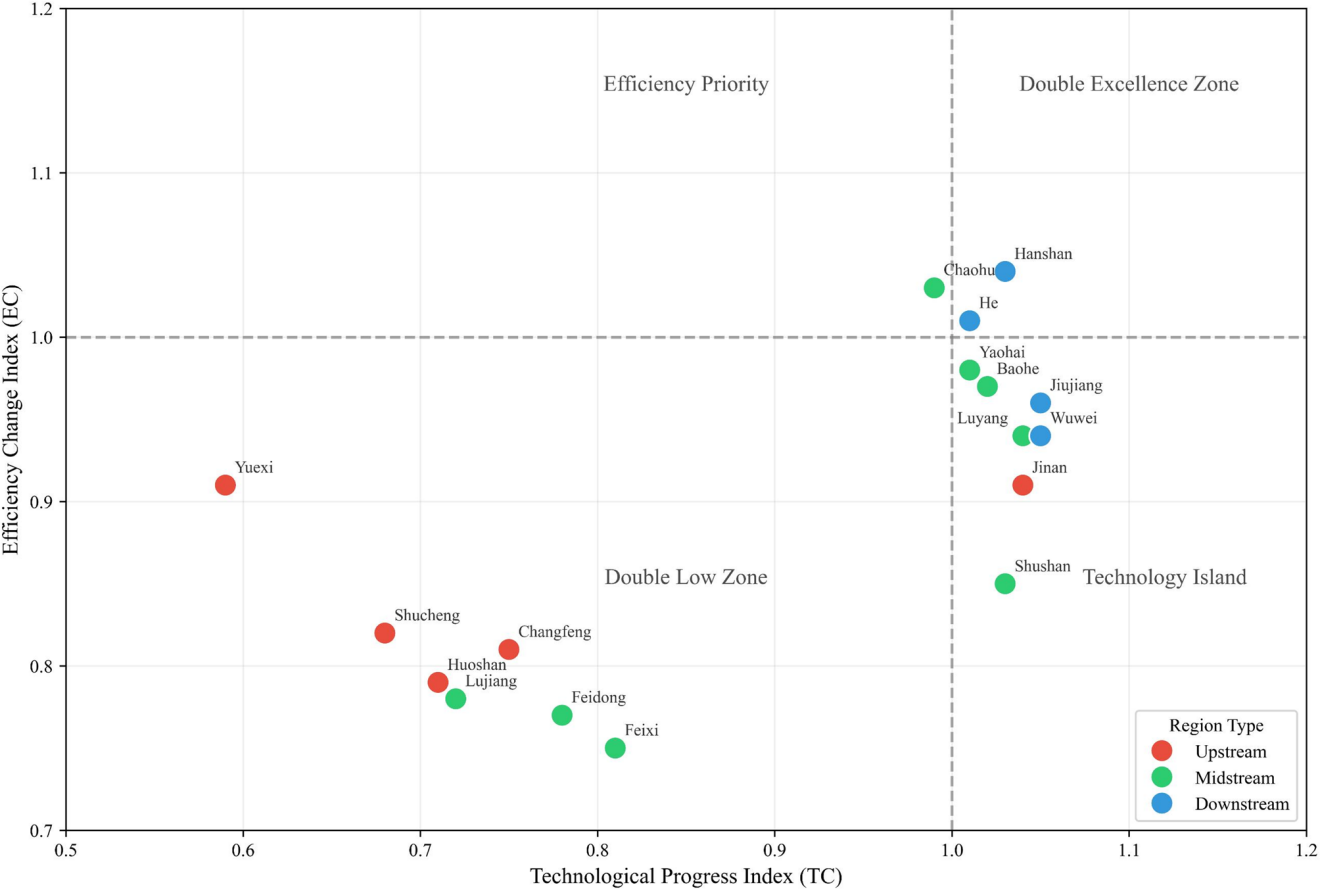
Distribution of TC-EC in counties and districts of the Chaohu Basin.

To visualise the collaborative status of technical and management capabilities across counties and districts, this paper plots efficiency value scatter plots based on $f_{x} = 0$ and $f_{x} = 1$. Due to space constraints, only the 2023 scatter plot is presented here; scatter plots for 2016–2022 are detailed in the appendix. As shown in Fig. 3 and Figs. 1–7 in the appendix, the coverage of the Double High Model Zones has expanded from 2016 to 2023.Core districts such as Baohe District and Yaohai District have consistently remained within this zone, with some districts exhibiting absolute efficiency approaching 1. This demonstrates the synergistic advantages of technological application and management efficiency, highlighting how certain regions have become benchmarks for enhancing basin efficiency. Nevertheless, a small number of districts and counties within the high-efficiency zones still possess scope for marginal optimisation, necessitating further exploration of potential in detailed management and technological refinement. Moreover, it was observed that scale-advantaged regions such as Chaohu City and He County, while leveraging economies of scale to narrow their potential for improvement, failed to achieve dual-high standards in absolute efficiency. This indicates insufficient synergy between the precision of technology transfer, the degree of management refinement, and operational scale. Future efforts must therefore strengthen the implementation of technologies and management adaptation to drive comprehensive efficiency gains and balanced development across diverse regional types within the basin.

#### Fluctuations in the efficiency of global governance

Based on the GML Index data from 17 districts and counties between 2017 and 2023, this study reveals that the efficiency of basin-wide governance exhibits significant fluctuations. By comprehensively examining the mean, minimum, maximum, and standard deviation, the dynamic evolution trajectory and fluctuation characteristics of governance efficiency can be elucidated.

As shown in Table 6 below, the overall trend indicates that the mean value of the GML Index follows a cyclical pattern of ‘policy effectiveness—efficiency decline—adjustment and recovery.’ The index mean reached its peak during the study period in 2018 (1.0640), coinciding closely with the implementation of strong intervention policies such as the Opinions on Accelerating the Development of a Green and Beautiful Chaohu Lake issued in June 2017 and the launch of the Ten Major Wetland Restoration Projects around Chaohu Lake in September of the same year. This demonstrates that concentrated policy initiatives can significantly enhance the overall management effectiveness of a river basin within a short timeframe. However, over the subsequent four years (2019–2022), the index mean declined steadily, falling below the efficiency threshold for the first time in 2022 (0.9833). This period coincided with policy consolidation and local pilot adjustments under the framework of the Comprehensive Green Development Master Plan for Chaohu Lake (issued December 2018). The establishment of the Chaohu Lake Expert Committee also prompted adjustments and reassessments of certain measures, leading to temporary efficiency fluctuations. Concurrently, major events such as the 2020 basin-wide floods and subsequent wetland flood storage operations may have impacted short-term agricultural activities and pollution loads. By 2023, the index mean had recovered to 1.0600, aligning with the accelerated implementation of the integrated protection and restoration project for ‘mountains, waters, forests, farmland, lakes and grasslands’ approved in May 2021, and the city-wide mobilisation conference on comprehensive Chaohu Lake management convened in March 2021. The latter reshaped policy momentum, enhancing synergies between technological innovation and management execution.

**Table 6:** Annual statistics for the GML index.

Year	Mean	Minimum value	Maximum value	Standard deviation
2017	0.9668	0.7398	1.0842	0.0758
2018	1.0640	0.8760	1.2892	0.1117
2019	1.0030	0.8236	1.1124	0.0705
2020	1.0308	0.9872	1.2340	0.0696
2021	1.0020	0.8601	1.0698	0.0433
2022	0.9833	0.8766	1.0125	0.0352
2023	1.0600	1.0000	1.3554	0.1014

Volatility is quantified through standard deviation. The standard deviations for 2018 and 2023 were 0.1117 and 0.1014 respectively, significantly higher than other years, indicating that governance efficiency divergence among counties and districts was most pronounced during these two periods. Extreme value analysis reveals a maximum of 1.2892 and a minimum of 0.8760 in 2018, highlighting uneven distribution of policy dividends within the basin—some districts achieved efficiency leaps while others lagged behind. In contrast, the standard deviations for 2021 and 2022 were lower (0.0433 and 0.0352), indicating that efficiency performance across districts and counties tended to converge during this phase, with the overall situation entering a period of adjustment and stagnation.

Stability and potential in efficiency can be discerned through changes in extreme values. The minimum value fluctuated upward from 0.7398 in 2017 to 1.0000 in 2023, indicating continuous improvement in efficiency-lagging counties within the basin and a gradual elevation of the overall efficiency floor. The maximum value fluctuated upward from 1.2892 in 2018 to 1.3554 in 2023, indicating that the basin’s ‘efficiency frontier’ continues to expand outward, with best practices in technological innovation and management optimization continually emerging.

Therefore, the governance efficiency of the Chaohu Lake basin does not exhibit linear improvement. Instead, it is driven by multiple factors such as policy cycles, technology diffusion, and management adaptability, showing distinct phased fluctuations and divergence characteristics. This dynamic feature suggests that while policy interventions may yield short-term results, they cannot guarantee sustainable efficiency. Establishing long-term governance mechanisms and deepening the synergy between technology and management are the keys to consolidating effectiveness.

#### Structural imbalance in technology and management

To investigate the respective contributions of technology and management to governance efficiency within the Chaohu Lake basin and their synergistic relationship, this study quantifies the specific values of technological progress (TC) and management efficiency (EC) along with their respective contributions, as detailed in Table 7.

**Table 7:** Technological progress and efficiency changes by district and county.

District	TC	EC	TC contribution(%)	EC contribution(%)	Core imbalance issue
Baohe	1.0002	1.0000	100.00	0.00	Technology-driven single-dimensional approach
Chaohu	0.9994	1.0274	− 6.14	106.14	Management of Subrogation
Feidong	1.0287	1.0000	100.00	0.00	Technology-driven single-dimensional approach
Feixi	1.0377	1.0078	161.91	− 61.91	Technical silos
Hanshan	1.0096	1.0010	281.67	− 181.67	Technical silos
He	1.0229	0.9928	− 1647.51	1747.51	Overcompensation in Management
Huoshan	1.0250	1.0000	100.00	0.00	Technology-driven single-dimensional approach
Jinan	1.0858	1.0312	130.61	− 30.61	Technical silos
Jiujiang	1.0233	1.0087	157.50	− 57.50	Technical silos
Lujiang	0.9991	1.0156	− 6.35	106.35	Management of Subrogation
Luyang	1.0002	1.0000	100.00	0.00	Technology-driven single-dimensional approach
Shucheng	1.0006	1.0275	3.29	96.71	Management of Subrogation
Shushan	0.9982	0.9933	21.86	78.14	Disparity in technical and managerial resources
Wuwei	1.0477	1.0425	83.62	16.38	Technology-led, management-coordinated
Yaohai	0.9983	1.0000	100.00	0.00	Technological lag and single-dimensional driving
Yuexi	1.0247	1.0092	160.38	− 60.38	Technical silos
Changfeng	1.0022	1.0000	100.00	0.00	Technology-driven single-dimensional approach

Such extreme values (e.g., He County: − 1647.51% and 1747.51%) indicate that a particular factor (e.g., management factors relative to technical factors) holds a dominant position; due to the decomposition into a multiplicative form, percentages may exceed 100%

To thoroughly investigate the contribution levels and synergistic relationship between technological progress and management efficiency in addressing agricultural non-point source pollution within the Chaohu Lake basin, this study calculated the technological progress index (TC) and management efficiency change index (EC) for each county based on GML index decomposition results. It further computed their respective contribution rates to total factor productivity (TFP) changes, as detailed in Table 7. By comparing the values of TC and EC and analyzing their contribution structures, several typical imbalance patterns were identified, revealing the structural bottlenecks of insufficient synergy between technology and management in the current process of enhancing basin governance efficiency.

As Table 7 indicates, the underlying causes of the imbalance between technological progress (TC) and managerial efficiency (EC) across districts and counties centre on core issues such as technological silos, mechanisms for technology-management coordination, and managerial compensation: He County’s technological contribution is negative, with excessive reliance on management compensating for technical shortcomings; Fei Xi County, Han Shan County, Jinan District, JiuJiang District, and YueXi County exhibit excessive technical contributions that have become isolated from the management system, forming technical silos; Chaohu City and Lujiang County exhibit negative technological contributions, relying on managerial compensation to offset technological shortcomings; Shucheng County demonstrates minimal technological contribution, with governance processes overly dependent on managerial efforts; Shushan District suffers from low technological and managerial efficiency, lacking effective systemic support; Wuwei City features technology-led governance with managerial coordination, yet exhibits imbalanced technological-managerial resource allocation. Yaohai District exhibits slight technological lag with single-dimensional drivers, where management fails to compensate; Baohe District, Feidong County, Huoshan County, Luyang District, and Changfeng County employ single-dimensional technological governance, with management gaps preventing the full realisation of technological value.

#### Coordination of technology and management with GML correlation

To investigate the correlation between the degree of technological-managerial coupling and coordination in the Chaohu Lake basin and the GML index, this study adopts the coupling coordination model to calculate the technological progress index and management efficiency index for 17 districts and counties, following the methodology of Wu et al^23^. The coupling coefficient C represents the degree of association between TC and EC, calculated by the formula:$C = 2 \times \sqrt {\left( {TC \times EC} \right)} /\left( {TC + EC} \right)$, The closer C approaches 1, the stronger the correlation between technology and management, and the better the coupling. The composite index reflects the overall level of TC and EC, This paper employs equal weighting, that is $T = 0.5 \times TC + 0.5 \times EC$; the coordination level $D = \sqrt {\left( {C \times T} \right)}$. Drawing upon the data characteristics of this study, this paper adopts the relative ranking method to classify coordination states, incorporating the coupled coordination degree grading approach proposed by He and Liu^24^. The high coordination zone (D > 1.030) indicates a high degree of synergy between technology and management, whereas the low coordination zone (D < 1.0) suggests a marked disconnect between the two.

Based on the coordination degree calculations, this paper plots a scatter diagram of the GML index against coordination degree, as detailed in Fig. 4. It is readily apparent from Fig. 4 that a strong positive correlation exists between coordination degree and the GML index. Overall, higher coordination degree indicates stronger synergistic effects between technology and management, thereby more effectively driving improvements in governance efficiency. Conversely, insufficient coordination significantly constrains enhancements in governance effectiveness, becoming a key bottleneck to efficiency gains.

#### County-level analysis

To investigate trends in efficiency across counties within the Chaohu Lake basin, this study employed the SBM-DDF-GML model to derive the GML indices for each county, as detailed in Table 8. Table 8 reveals a pronounced polarization in governance effectiveness across the Chaohu Lake basin. Districts such as Jinan and Jiujiang have achieved leapfrog improvements, emerging as high-efficiency leaders. Conversely, districts like Shushan face stagnation or decline, making them key targets for improvement. As shown in Table 8, the GML indices across districts and counties from 2017 to 2023 exhibit significant variation, primarily manifested in the magnitude of annual fluctuations and overall change. In terms of volatility, Huoshan County recorded 1.2892 in 2018, yet plummeted to 0.8236 in 2019, representing an annual deviation exceeding 0.46. Jin’an District registered 1.0037 in 2017, rising to 1.3554 by 2023, with a difference between the initial and final values reaching 0.35. Jiujiang District stood at 0.7398 in 2017, reaching 1.2584 by 2023, reflecting a seven-year change of 0.5186. Regarding stability, Luyang District consistently maintained a value of 1.0 from 2019 to 2023, with annual fluctuations approaching zero. He County’s annual values ranged between 0.9598 and 1.0226, with the maximum annual variation being merely 0.06. Regarding change patterns, only Chaohu City and Shushan District experienced efficiency declines, while the remaining 15 districts and counties all improved. However, the magnitude of improvement varied significantly, ranging from 0.0024 to 0.5186, clearly illustrating the differentiated characteristics of governance effectiveness across these administrative units.

**Table 8:** GML index and efficiency changes by district and county.

District	2017	2018	2019	2020	2021	2022	2023	Mean	Change	Type of change
Baohe	1	1	0.9707	1.0302	1	0.9841	1.0161	1.0002	0.0161	Enhancement
Chaohu	1.0056	1.0491	0.9996	0.9986	1.0081	1.0072	1.0035	1.0102	−0.0021	Decline
Feidong	0.9063	1.2878	0.9420	0.9872	0.9726	1.0074	1.0974	1.0287	0.1911	Enhancement
Feixi	0.9911	1.1529	0.9717	1.0128	0.9923	1.0125	1.0297	1.0233	0.0385	Enhancement
Hanshan	0.9789	1.0374	1.0003	1.0009	0.9969	0.9990	1.0105	1.0034	0.0317	Enhancement
He	0.9598	1.0226	1.0009	1.0002	1.0025	0.9959	1.0085	0.9986	0.0486	Enhancement
Huoshan	0.9956	1.2892	0.8236	1.0385	1.0095	0.9941	1.0245	1.0250	0.0289	Enhancement
Jinan	1.0037	0.9339	1.1047	0.9894	1.0698	1.0028	1.3554	1.0657	0.3518	Enhancement
Jiujiang	0.7398	1.0562	1.0777	1.1874	0.8601	0.9239	1.2584	1.0148	0.5186	Enhancement
Lujiang	0.9612	1.1524	0.9901	1.0268	0.9999	0.9569	1.0157	1.0147	0.0544	Enhancement
Luyang	0.9656	1.0357	1	1	1	1	1	1.0002	0.0344	Enhancement
Shucheng	1.0210	0.9432	1.1124	1.0072	1.0134	1.0015	1.0242	1.0176	0.0032	Enhancement
Shushan	1.0842	0.8760	0.9449	1.0079	1.0512	0.9684	1.0106	0.9919	−0.0736	Decline
Wuwei	0.9380	1.1087	1.1011	1.2340	1	0.8766	1.1407	1.0570	0.2028	Enhancement
Yaohai	1	0.9971	0.9967	0.9984	1.0043	0.9893	1.0024	0.9983	0.0024	Enhancement
Yuexi	1.0183	1.0423	1.0232	0.9928	1.0125	0.9966	1.0223	1.0154	0.0040	Enhancement
Changfeng	0.8673	1.1033	0.9918	1.0119	1.0414	1	1	1.0022	0.1327	Enhancement

Using the mean GML index value from 2017 to 2023 as the core classification criterion, a three-tier classification standard is established: Low-efficiency zones are defined as GML mean < 1.0, indicating long-term governance efficiency below the benchmark with overall suboptimal performance; Medium-efficiency zones are defined as 1.0 ≤ GML mean < 1.02, signifying long-term efficiency meeting standards but without establishing significant competitive advantage; High-efficiency zones are defined as GML mean ≥ 1.02, indicating long-term efficiency significantly exceeding benchmarks and governance effectiveness at the leading level within the basin.

The classification results are as follows. The high-efficiency zone comprises Feidong County, Feixi County, Huoshan County, Wuwei City, and Jinan District, where all districts and counties recorded average values exceeding 1.02, demonstrating particularly outstanding results in technology implementation and management coordination. The medium-efficiency zone encompasses Baohe District, Chaohu City, Hanshan County, Lujiang County, Luyang District, Shucheng County, Jiujiang District, Yuexi County, and Changfeng County, where overall efficiency meets standards but exhibits annual fluctuations; The low-efficiency zone comprises He County, Yaohai District, and Shushan District, where long-term governance effectiveness requires urgent improvement.

### Analysis of regional heterogeneity

To provide tailored zonal management strategies for agricultural non-point source pollution in the Chaohu Lake basin and address the challenge of uneven governance across the entire region, this study divides the basin into three major zones—upper, middle, and lower reaches—based on water flow direction. Utilising data on input–output slack values, technological progress, and management efficiency, it systematically explores the regional heterogeneity of the Chaohu Lake basin, thereby providing empirical conclusions for differentiated regional governance. Although the efficiency evaluation model treats each county and district as an independent decision-making unit, this paper employs a zoned integration analysis based on water flow patterns to indirectly reveal the spatial correlations and potential transmission mechanisms underlying pollution control characteristics within the basin. It systematically presents the heterogeneous features of the upper, middle, and lower reaches of the Chaohu Lake basin, providing empirical conclusions for regionally differentiated governance.

#### Input–output slack characteristics

To investigate the regional characteristics of input–output patterns across the upper, middle, and lower reaches of the Chaohu River Basin, this study organised data based on the 2016–2023 slack variable results for each county and indicator. This yielded the mean input and output slack values for the three regions, presented respectively in Tables 9 and 10.

**Table 9:** Regional mean input slack.

Region	Labor input	Land input	Machinery input	Irrigation input	Pesticide input	Fertilizer input
Upstream	0.1395	0.1113	0.1426	0.1110	0.1728	0.1243
Midstream	0.1290	0.0642	0.0625	0.1413	0.1038	0.1010
Downstream	0.1182	0.1149	0.0786	0.1881	0.1891	0.2846

**Table 10:** Regional output slack mean.

Region	Expected output	Undesirable output—agricultural carbon emissions	Undesirable output—total emissions of nitrogen and phosphorus
Upstream	0.0376	0.1675	0.1110
Midstream	0.0097	0.1836	0.0663
Downstream	0.0089	0.1650	0.1167

As shown in Tables 9 and 10, the mean slack for labor input in the upstream region stands at 0.1395, exceeding that of both downstream and midstream areas. This indicates relatively low efficiency in agricultural labor resource allocation within the upstream region, suggesting a degree of labor redundancy. The mean slack for machinery input is significantly higher than in downstream and midstream regions, reflecting a mismatch between local agricultural machinery deployment and actual operational requirements, resulting in idle machinery resources. The relatively high mean slack for pesticide inputs indicates a greater reliance on chemical pesticides for pest and disease control. Although the slack for fertilizer inputs is low, there remains scope for optimisation. In terms of output, while the upstream region achieved some success in actual agricultural output value, it exhibited the highest mean deviation from expected output. This indicates the region has not attained optimal production conditions, with output falling short of expectations. Among undesirable output indicators, the slack mean for agricultural carbon emissions stands at 0.1675, while that for total nitrogen and phosphorus emissions is 0.1110. This indicates that non-point source pollution remains at a certain level, demonstrating that agricultural production exerts a degree of environmental impact. Consequently, local authorities should prioritise the development of green agriculture.

For the midstream region, the mean land input slack is significantly lower than that of the upstream and downstream regions. This indicates that land transfer policies are well-established and land is managed with a high degree of concentration in this area, enabling rational planning and efficient utilisation of land resources. The relatively low mean machinery input slack suggests that agricultural machinery allocation aligns well with operational requirements. The lowest mean pesticide input slack indicates proactive adoption of scientific methods such as biological control in pest and disease management, reducing reliance on chemical pesticides. In terms of output, the agricultural industry in the midstream region is not particularly prominent, and the value-added of agricultural products is not high. However, input allocation is relatively reasonable, and under ideal conditions, output is relatively efficient. The mean slack coefficient for agricultural carbon emissions was higher than that of upstream and downstream sectors, indicating significant energy consumption during agricultural production in this region. The mean slack coefficients for total nitrogen and total phosphorus emissions were the lowest. Combined with fertilizer application data, this demonstrates that scientifically sound fertilizer management practices effectively controlled total nitrogen and phosphorus emissions, thereby reducing water pollution and playing a positive role in protecting the water environment of Lake Chaohu.

For the downstream region, the mean labor input slack is lower than that of the upstream and midstream regions, indicating relatively more efficient allocation of labor resources in this area. The mean irrigation input slack is higher than that of the upstream and midstream regions, suggesting that downstream areas may suffer from excessive irrigation infrastructure development or inefficient irrigation practices, leading to wastage of water resources and irrigation facilities. The mean slack for pesticide inputs remains at a relatively high level, reflecting a greater reliance on chemical pesticides for pest and disease control. The mean slack for fertilizer inputs stands at 0.2846, significantly higher than in the upper and middle reaches, indicating a more severe problem of excessive fertilizer application in the downstream region. In terms of output, the region demonstrates suboptimal economic efficiency in agricultural production, though under ideal conditions yields are relatively efficient. The mean slack values for total nitrogen and total phosphorus emissions exceed those of the upper and middle reaches. When combined with the mean slack values for fertilizer inputs, this indicates that excessive fertilizer application is the primary cause of elevated total nitrogen and total phosphorus emissions.

#### Synergistic characteristics of technological progress and management efficiency

To systematically reveal the synergistic differences in technological progress and management efficiency during agricultural non-point source pollution control across the upper, middle, and lower reaches of the Chaohu Lake basin, Building upon prior analyses, this study conducts descriptive statistical analysis on key indicators including the Technological Progress Index (TC), the Evolution of Control Efficiency Index (EC), their contribution structures, the proportion of high-efficiency zones, and resource waste rates across each region, as detailed in Table 11. The aim is to delve into the distinct manifestations of the synergistic relationship between technology and management across different regions, thereby providing data support for formulating subsequent targeted governance strategies tailored to specific zones and categories.

**Table 11:** Upstream-midstream-downstream indicator comparison.

Indicator	Upstream mean	Midstream mean	Downstream mean
GML Index (2017–2023)	0.982	1.010	1.024
TC contribution (%)	− 35.6	68.4	121.8
EC contribution (%)	135.6	31.6	− 21.8
High-efficiency zone coverage (%)	68.2	88.5	95.2
Resource wastage rate (%)	68.0	32.5	22.5

To visually illustrate the regional variations in technical capacity (TC) and executive capacity (EC) across the upper, middle, and lower reaches of the Chaohu Lake basin, alongside their degree of synergy, this paper has mapped the distribution of governance technologies and management approaches across the basin’s counties and districts, as shown in Fig. 5. Combining Table 11 with Fig. 5 reveals that upstream counties predominantly cluster within the ‘dual-low zone’ characterised by TC < 1 and EC < 1. This reflects their predominantly agricultural nature and the governance challenges posed by dispersed non-point source pollution. A pronounced gap in technology transfer is evident, with inefficient governance leading to persistent pollution inputs towards midstream and downstream areas. Midstream counties exhibit transitional characteristics, with some edging into the ‘efficiency-first zone’ or the periphery of the ‘dual-excellence zone’. However, their overall remediation efficiency remains highly volatile. Although their mean GML index outperforms upstream areas, policy implementation lacks stability. Downstream counties predominantly cluster within ‘technological isolation zones’, clearly illustrating the contradiction of ‘high technological investment coupled with lagging management’. Ultimately, differing governance foundations and approaches across upstream, midstream and downstream sectors have led to a cascade of transmission-related contradictions: upstream sectors dragging down progress, midstream sectors destabilising, and downstream sectors operating in vain.

#### Further exploration

To explore the primary causes of regional heterogeneity, this paper examines three aspects: upstream, midstream, and downstream. Regarding the upstream issue of ‘low technology and low management,’ the fragmented terrain and scattered farmland in upstream mountainous areas (Yuexi, Huoshan) make it difficult for large agricultural machinery to operate, objectively limiting the adoption of mechanized technology. Additionally, as key ecological functional zones, industrial development is restricted. Local fiscal capacity for matching subsidies for green technologies is weak. The outflow of young and middle-aged labor leaves behind an older farming population with insufficient willingness to adopt new technologies and limited learning capacity, creating a ‘low-technology lock-in.’ Shucheng County and Jinan District, characterized by high hill coverage and poor soil water and nutrient retention, further exacerbate input excess.

Regarding the ‘unstable policy implementation’ in the midstream, these areas (particularly Hefei’s urban core and near-suburbs) face multiple conflicting policy objectives, ensuring urban agricultural supply while rapidly reducing agricultural non-point source pollution, coupled with the reform mandate of serving as a ‘pilot zone’ for the Chaohu Lake Ecological Demonstration Area. Local governments frequently adjust priorities across phases (e.g., high-pressure implementation during the 2017 intensive campaign, policy evaluation and adjustment from 2019 to 2021), while some remediation projects rely on central and provincial special funds, causing efficiency fluctuations. This phenomenon, termed the ‘policy experimentation-normalization tension’ in watershed governance literature^22^, This study provides empirical evidence for this theory from an efficiency perspective.

The study reveals an overinvestment phenomenon, particularly pronounced in pesticide and labor surpluses in upstream regions. Such surpluses may exacerbate soil disturbance and weaken surface cover, thereby elevating erosion risks and the associated pollutant migration flux^12,13^.

## Conclusions and recommendations

This study employs panel data from 17 counties (districts) within the Chaohu Lake basin spanning 2016–2023. It utilises the SBM-DDF model to conduct a static measurement of agricultural non-point source pollution control efficiency. Building upon this foundation, it further employs the Global Malmquist-Luenberger (GML) index to perform a dynamic decomposition of this efficiency. Additionally, regional heterogeneity is examined across the upper, middle, and lower reaches of the Chaohu Lake basin. Research findings reveal: (1) Agricultural inputs within the Chaohu Lake basin exhibit widespread redundancy, with excessive application of chemical fertilizers and pesticides constituting the core issue, alongside low utilisation efficiency of labor, machinery and irrigation resources. (2) Basin-wide governance efficiency demonstrates cyclical fluctuations characterised by ‘policy effectiveness – efficiency decline – adjustment recovery’, with weak synergies between technological advancement and management efficiency severely constraining overall governance efficacy. (3) Significant regional heterogeneity exists: upstream areas exhibit high redundancy in labor, machinery, and pesticide inputs alongside dual deficiencies in technology and management; midstream regions demonstrate efficient land use and the lowest reliance on chemical pesticides, yet suffer from unstable policy implementation; downstream areas face prominent redundancy in fertilizer and irrigation, coupled with structural imbalances in technology and management. Based on these findings, the following recommendations are proposed:

1. Implement a targeted strategy to reduce excess capacity based on slack variables. To address over-input, focus on regulating fertilizer and pesticide application in areas with high excess capacity at the county level. Promote ecological agricultural technologies such as soil testing and formula fertilization, green pest control, and organic fertilizer substitution to reduce pollutant emission intensity at the source. Simultaneously, incorporate precision agriculture technologies like drone variable-rate fertilization and intelligent irrigation decision systems into the promotion plan. Implement a ‘one county, one policy’ approach to excess reduction projects. In key fertilizer areas (Xiaoxiang and Wuwei counties downstream), comprehensively promote side-deep fertilization and water-fertilizer integration. In high pesticide dependency areas (Shucheng and Yuexi counties upstream), establish intelligent pest and disease monitoring stations, incorporating green pest control coverage into township performance evaluations. In irrigation-excess areas (Hangshan and Jiujiang counties downstream), initiate water-saving renovations in irrigation districts and install water measurement facilities in canal systems. In mechanization-idle areas (upstream Huoshan, Jinan), implement a ‘custodial + scheduling’ model through agricultural machinery cooperatives.
2. Establish an efficiency-oriented long-term governance mechanism and deepen the integration of technology and management. Incorporate indicators such as the GML Index and coordination level into the policy evaluation system. Implement ecological compensation and dynamic supervision mechanisms based on governance efficiency. Simultaneously incentivize districts and counties to continuously enhance governance effectiveness through a dual strategy of oversight and encouragement. Build a cross-departmental data sharing and response platform to promote synergy between technological innovation and management mechanisms, avoiding ‘island effects’ and over-regulation. Pilot the ‘Technology-Management Synergy Enhancement’ initiative in Feixi County (a typical technical silo) and He County (a typical management compensation case). Feixi County reduces machinery idling through scaled operations; He County strengthens fertilizer reduction technology adoption via its technology promotion network.
3. Implement zoned collaborative governance strategies. Address regional heterogeneity across upstream, midstream, and downstream areas through differentiated approaches. Upstream: Establish provincial green agriculture compensation funds providing direct household subsidies based on pesticide reduction volumes and the expansion of contour planting on sloping farmland. Simultaneously support subsidies for biological pesticides and ecological ditch construction, aiming for a 10% reduction in upstream pesticide usage within several years compared to 2023 levels. In the midstream, implement a dual assessment system combining the River and Lake Chief System with efficiency targets, linking cross-section water quality compliance rates to the GML Index within jurisdictions. Establish a technical pilot base in the Lake Chaohu Ecological Demonstration Zone. Downstream, create irrigation district water user associations, linking smart irrigation decision systems to water quota trading. Farmers meeting water conservation targets per mu will receive economic subsidies. Hefei City will spearhead the establishment of a basin-level governance joint conference. A shared responsibility policy for water quality improvement will be implemented at key monitoring sections: if upstream water quality exceeds targets, downstream areas compensate upstream; if it falls below targets, upstream areas compensate downstream. This creates a closed-loop incentive mechanism of ‘shared responsibility and shared benefits’.

## Limitations and future prospects

This study systematically evaluates agricultural non-point source pollution control in the Chaohu Lake basin from a management efficiency perspective, yet certain limitations remain. First, the research focuses on relative efficiency assessment without integrating natural hydrological process models. Secondly, research based on observational panel data struggles to establish a rigorous causal relationship with relevant policies. Finally, while the county-level analysis units based on administrative divisions align with management practices, they fall short in quantifying spatial spillover effects within the basin. Future research may explore three directions, First, integrating efficiency assessment models with distributed hydrological models like SWAT to establish a comprehensive evaluation system linking ‘management efficiency-process simulation-ecological response.’ Second, develop methods like spatial network DEA to embed pollutant transport relationships within river networks into efficiency models, thereby more precisely identifying cross-regional collaborative governance pathways. Thirdly, more robust testing methods such as spatial DEA and regression tests can be employed to enhance the reliability of conclusions.

Constrained by current methodologies, spatial autocorrelation was not incorporated into the model; future work could integrate spatial econometric DEA or hydrological models for more refined spatial correlation analysis. Additionally, the time span of panel data could be extended to capture longer-term policy effects. Future research should explore these directions in greater depth.

## Supplementary Information

Below is the link to the electronic supplementary material.

Supplementary Material 1

## Data Availability

The data that support the findings of this study are available from the corresponding author upon reasonable request.

## References

[1] Carpenter, S. R. et al. Non-point pollution of surface waters with phosphorus and nitrogen. *Ecol. Appl.* **8**(3), 559–568 (1998).

[2] Luo, M. et al. Evaluation of agricultural non-point source pollution: A review. *Water Air Soil Pollut.* **234**(10), 657 (2023).

[3] Zhou, H. & Gao, C. Assessing the risk of phosphorus loss and identifying critical source areas in the Chaohu Lake watershed, China. *Environ. Manage.* **48**(5), 1033–1043 (2011). 21882000 10.1007/s00267-011-9743-z

[4] Jiang, T., Wang, M., Zhang, W., Zhu, C. & Wang, F. A comprehensive analysis of agricultural non-point source pollution in China: Current status, risk assessment and management strategies. *Sustainability* **16**(6), 2515 (2024).

[5] Qiu, W., Zhong, Z. & Li, Z. Agricultural non-point source pollution in China: Evaluation, convergence characteristics and spatial effects. *Chin. Geogr. Sci.* **31**(3), 571–584 (2021).

[6] Wang, M. et al. Current situation of agricultural non-point source pollution and its control. *Water Air Soil Pollut.* **234**(7), 471 (2023).

[7] Sun, B. et al. Agricultural non-point source pollution in China: Causes and mitigation measures. *Ambio* **41**(4), 370–379 (2012). 22311715 10.1007/s13280-012-0249-6 PMC3393061

[8] Hussain, F. et al. Agricultural non-point source pollution: Comprehensive analysis of sources and assessment methods. *Agriculture* **15**(5), 531 (2025).

[9] Xu, H., Tan, X., Liang, J., Cui, Y. & Gao, Q. Impact of agricultural non-point source pollution on river water quality: Evidence from China. *Front. Ecol. Evol.* **10**, 858822 (2022).

[10] Tone, K. A slacks-based measure of efficiency in data envelopment analysis. *Eur. J. Oper. Res.* **130**(3), 498–509 (2001).

[11] Zhu, K. W. et al. Coupling ITO3dE model and GIS for spatiotemporal evolution analysis of agricultural non-point source pollution risks in Chongqing in China. *Sci. Rep.* **11**(1), 4635 (2021). 33633279 10.1038/s41598-021-84075-2 PMC7907261

[12] Wang, Z. et al. Mechanical terracing regulates soil physicochemical properties and infiltration processes in the Loess Hilly Region of China. *Land. Degrad. Dev.* **35**(9), 3181–3190 (2024).

[13] Yang, D., She, D., Fang, N., Huang, X. & Shi, Z. Hydrological connectivity influences soil erosion and SOC loss on vegetation restoration slopes. *Soil. Tillage. Res.* **257**, 106991 (2026).

[14] Fukuyama, H. & Weber, W. L. A directional slacks-based measure of technical inefficiency. *Socio-Econ. Plan. Sci.* **43**(4), 274–287 (2009).

[15] Oh, D. H. A global Malmquist-Luenberger productivity index. *J. Prod. Anal.* **34**(3), 183–197 (2010).

[16] Khan, S. A. M. N., Ramli, R. & Baten, M. D. A slack based enhanced DEA model with undesirable outputs for rice growing farmers efficiency measurement. *Int. J. Supply Chain Manag.* **7**(1), 194–200 (2018).

[17] Hu, Y., Zhang, K., Hu, N. & Wu, L. Review on measurement of agricultural carbon emission in China. *Chin. J. Eco-Agric.* **31**(2), 163–176 (2023).

[18] Wang, T. et al. Integrated assessment of anthropogenic carbon, nitrogen, and phosphorus inputs: A Panjin City case study. *Water* **17**(20), 2962 (2025).

[19] Lai, Y., Yang, H., Qiu, F., Dang, Z. & Luo, Y. Can rural industrial integration alleviate agricultural non-point source pollution? Evidence from rural China. *Agriculture* **13**(7), 1389 (2023).

[20] West, T. O. & Marland, G. A synthesis of carbon sequestration, carbon emissions, and net carbon flux in agriculture: Comparing tillage practices in the United States. *Agric. Ecosyst. Environ.* **91**(1–3), 217–232 (2002).

[21] Tang, Y., Zhao, X. & Jiao, J. Ecological security assessment of Chaohu Lake Basin of China in the context of River Chief System reform. *Environ. Sci. Pollut. Res.* **27**(3), 2773–2785 (2020). 10.1007/s11356-019-07241-0 31836980

[22] Kong, J., Liu, Y., Li, J. & Gong, H. Diagnosis of performance and obstacles of integrated management of three-water in Chaohu Lake Basin. *Water* **17**(14), 2135 (2025).

[23] Wu, W., Liang, Z., Zhang, Q. & Zhang, H. Coupling relationships and synergistic mechanisms between technology management capability and technological capability in product innovation: A simulation study. *Technol. Anal. Strateg. Manag.* **32**(9), 1098–1112 (2020).

[24] He, X. & Liu, W. Coupling coordination between agricultural eco-efficiency and urbanization in China considering food security. *Agriculture* **14**(5), 781 (2024).

