# Supplementary material for: Spatio-temporal evolution and regional heterogeneity in the efficiency of agricultural non-point source pollution control within the Chaohu Lake Basin
Source: Sci Rep. 2026 Mar 31;16:15244. doi: 10.1038/s41598-026-45974-4 (PMC13181029; doi:10.1038/s41598-026-45974-4)
Supplement: Supplementary file 1 — Supplementary Material 1 [file 41598_2026_45974_MOESM1_ESM.docx]

# **Appendix**

**Appendix 1 Correlation of Paired Samples for Slack Variables**

| **pairing** | **Variable** | **Relevance** | **Significance** |
| --- | --- | --- | --- |
| Pairing 1 | X1-0 & X1-1 | .999 | .000 |
| Pairing 2 | X2-0 & X2-1 | 1.000 | .000 |
| Pairing 3 | X3-0 & X3-1 | .999 | .000 |
| Pairing 4 | X4-0 & X4-1 | 1.000 | .000 |
| Pairing 5 | X5-0 & X5-1 | 1.000 | .000 |
| Pairing 6 | X6-0 & X6-1 | 1.000 | .000 |
| Pairing 7 | Y1-0 & Y1-1 | .977 | .000 |
| Pairing 8 | Z1-0 & Z1-1 | 1.000 | .000 |
| Pairing 9 | Z2-0 & Z2-1 | 1.000 | .000 |

**Appendix 2 GML Index Pairing Sample Correlation**

| **pairing** | **Variable** | **Relevance** | **Significance** |
| --- | --- | --- | --- |
| Pairing 1 | GML-0 & GML-1 | .905 | .000 |
| Pairing 2 | TC-0 & TC-1 | .962 | .000 |
| Pairing 3 | EC-0 & EC-1 | .959 | .000 |

**Appendix 3 Abbreviations Explained**

| **Full name** | **Abbreviated** | **Full name** | **Abbreviated** |
| --- | --- | --- | --- |
| Baohe District | Baohe | Lujiang County | Lujiang |
| Chaohu City (county-level) | Chaohu | Luyang District | Luyang |
| Feidong County | Feidong | Shucheng County | Shucheng |
| Feixi County | Feixi | Shushan District | Shushan |
| Hanshan County | Hanshan | Wuwei City(county-level) | Wuwei |
| He County | He | Yaohai District | Yaohai |
| Huoshan County | Huoshan | Yuexi County | Yuexi |
| Jinan District | Jinan | Changfeng County | Changfeng |
| Jiujiang District | Jiujiang |  |  |


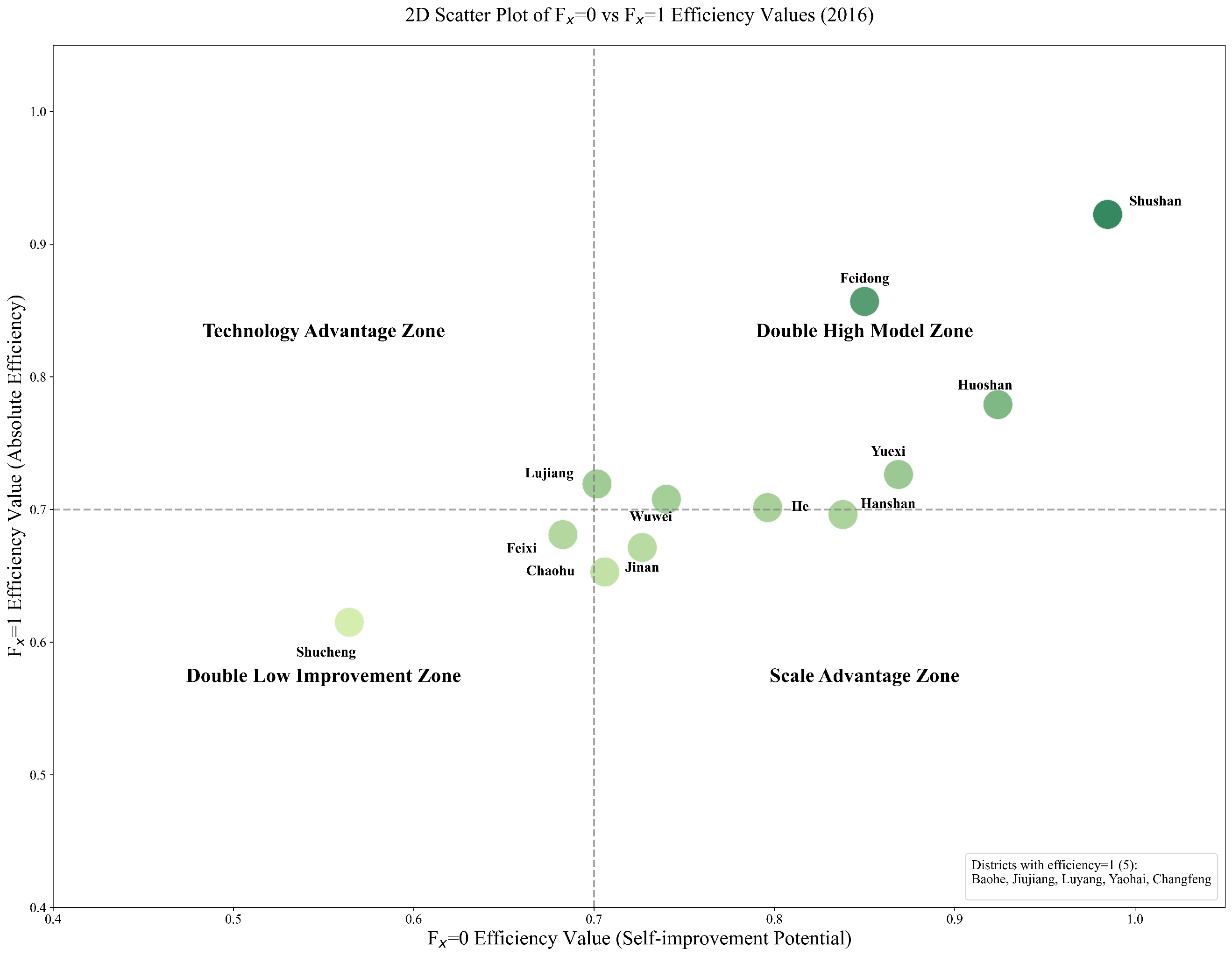


**Figure 1 2016 Efficiency Value Two-Dimensional Scatter Plot**


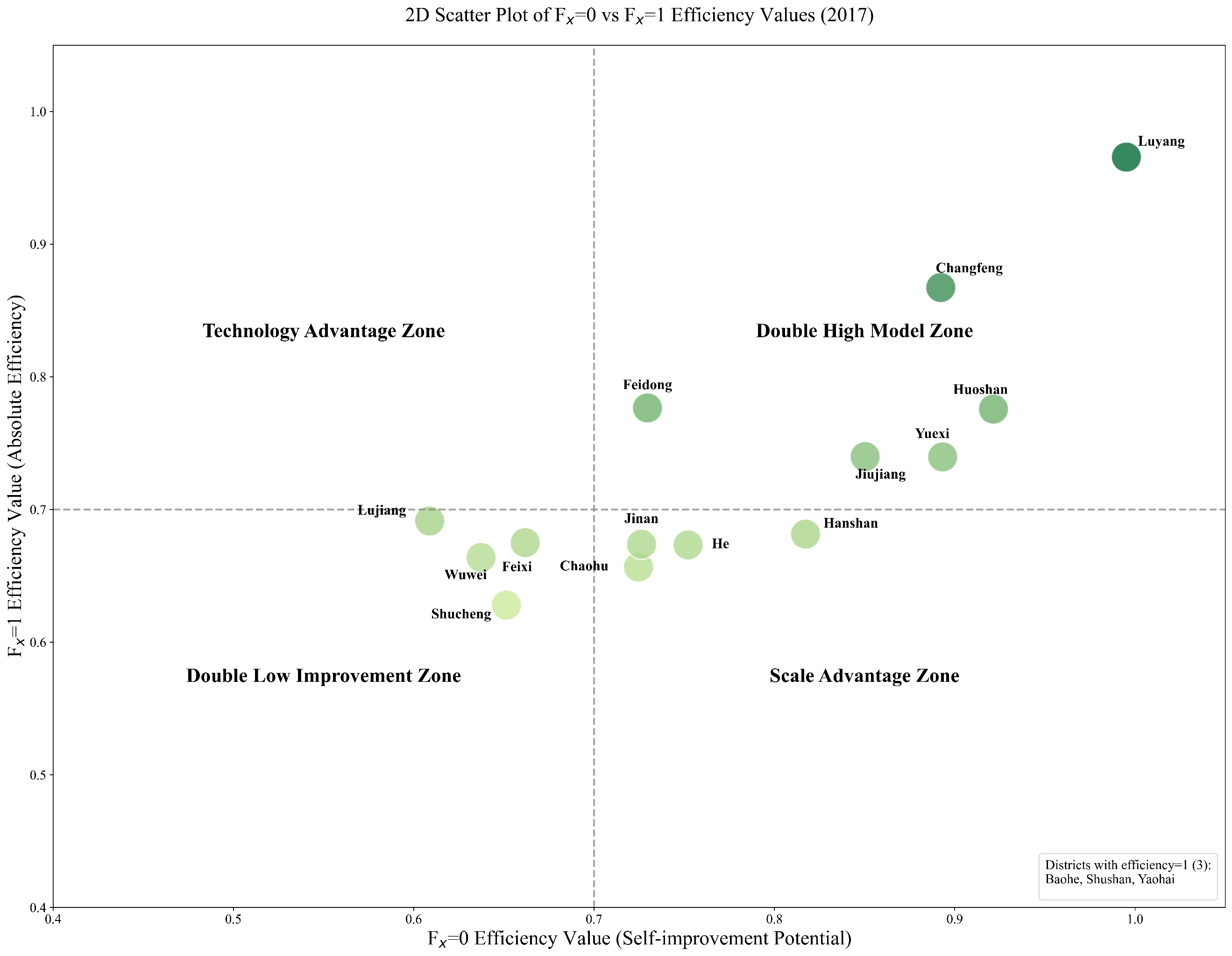


**Figure 2 2017 Efficiency Value Two-Dimensional Scatter Plot**


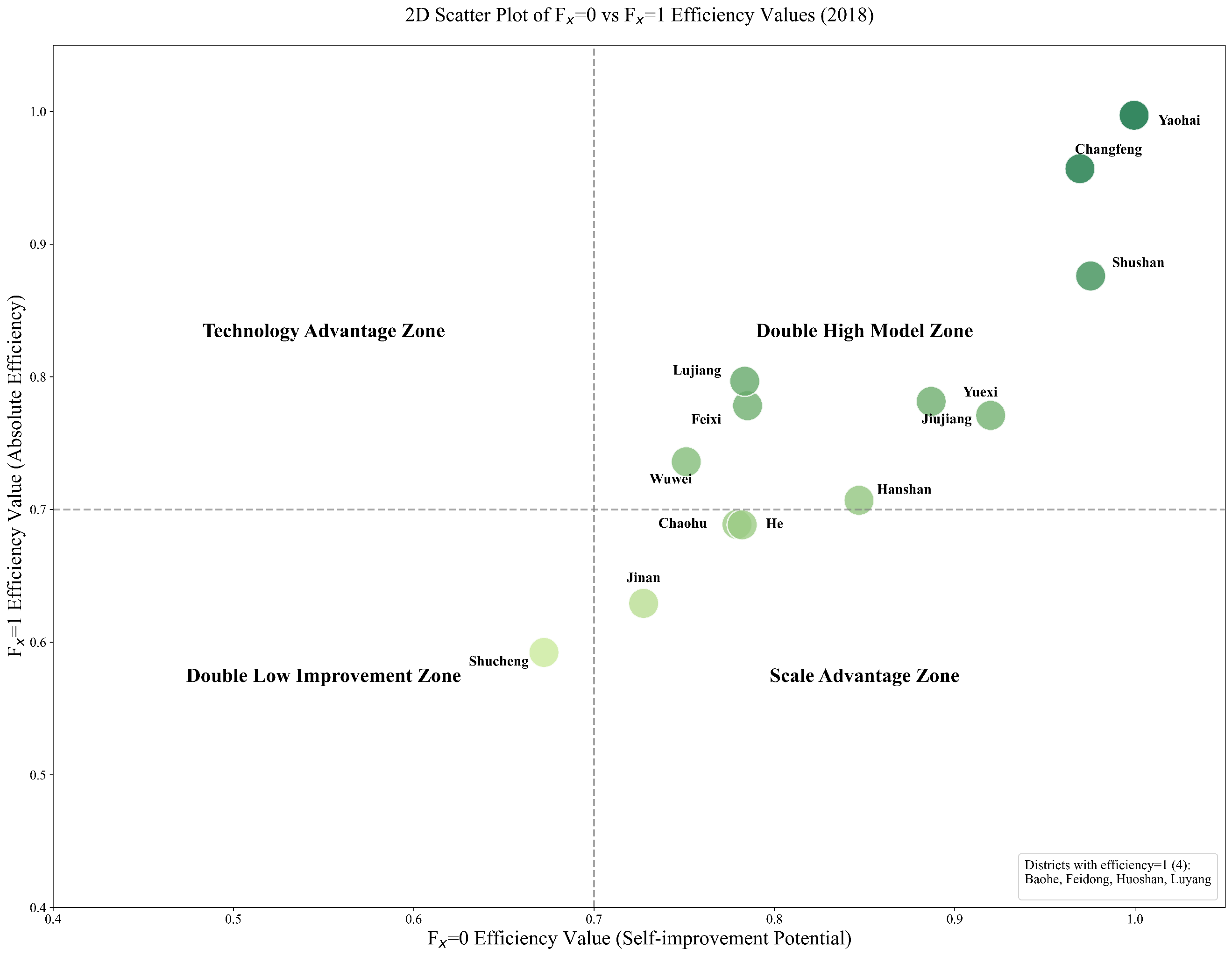


**Figure 3 2018 Efficiency Value Two-Dimensional Scatter Plot**


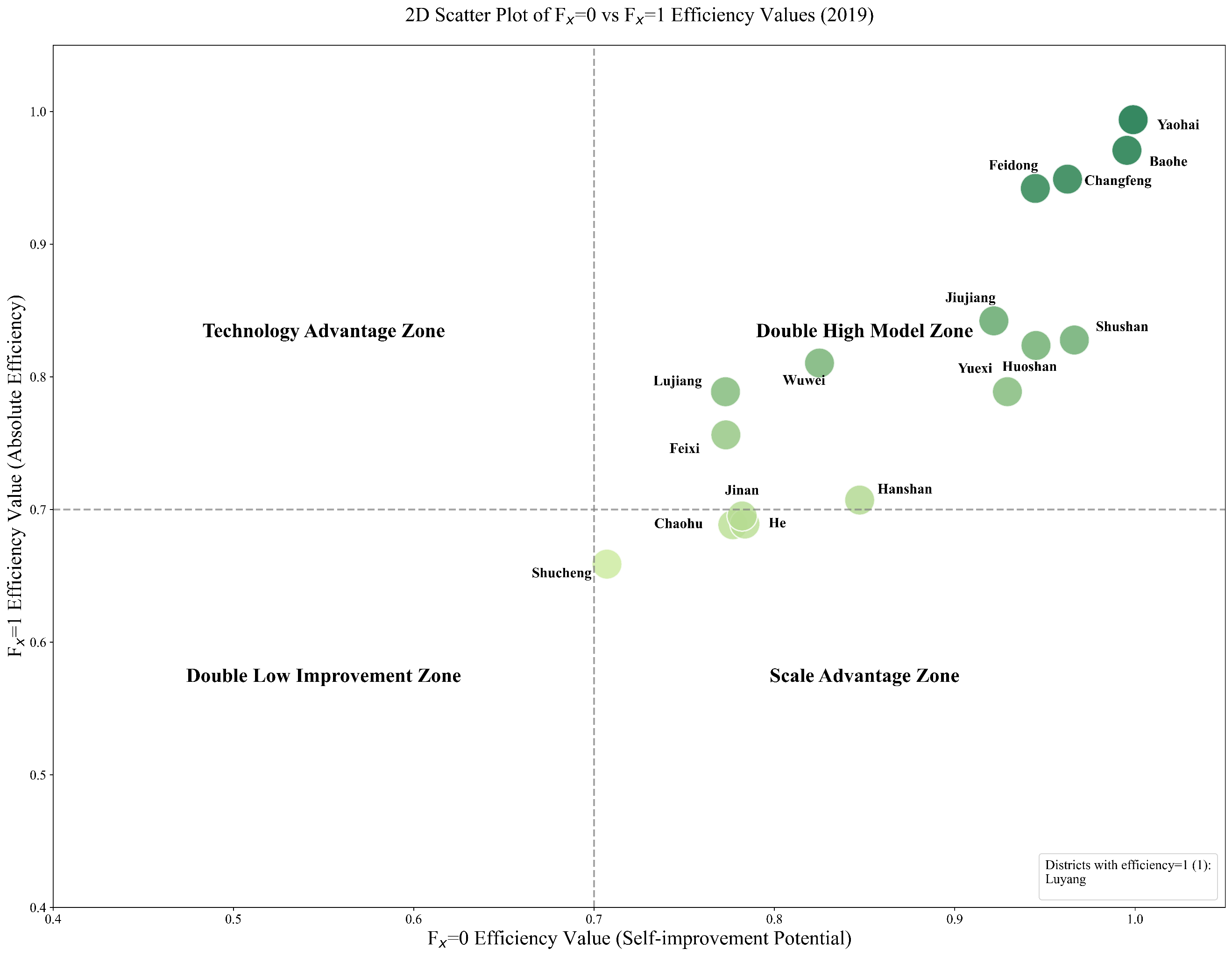


**Figure 4 2019 Efficiency Value Two-Dimensional Scatter Plot**


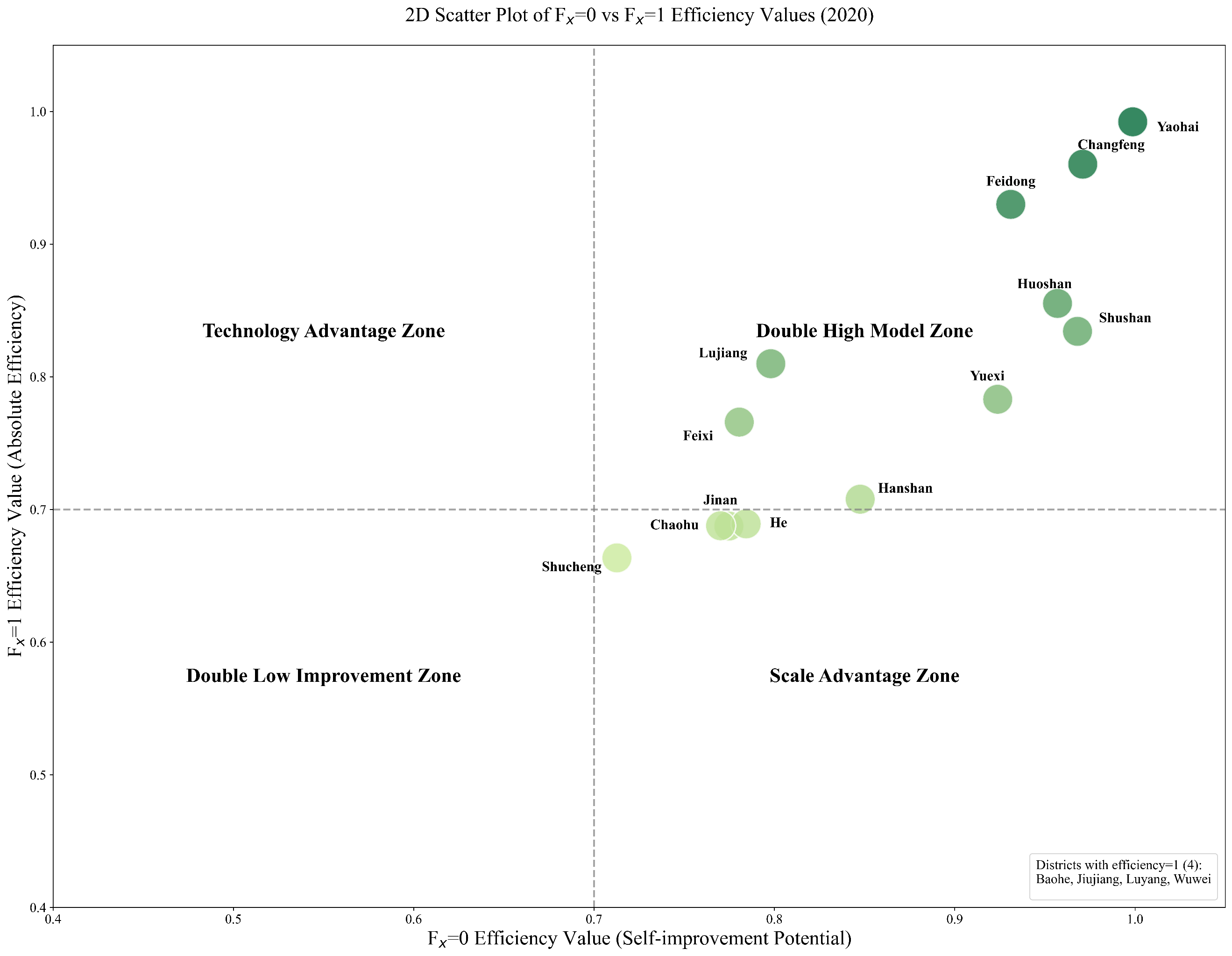


**Figure 5 2020 Efficiency Value Two-Dimensional Scatter Plot**


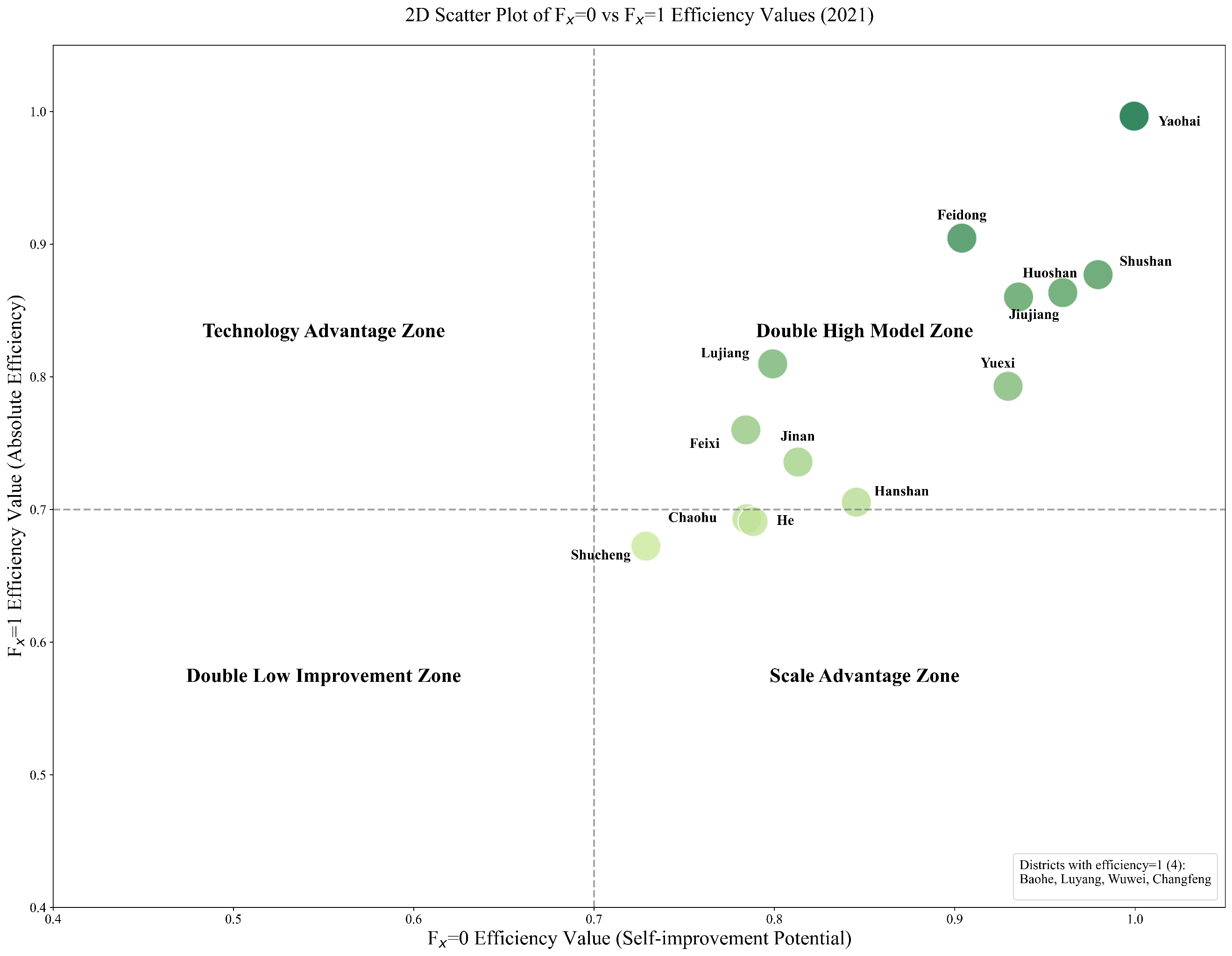


**Figure 6 2021 Efficiency Value Two-Dimensional Scatter Plot**


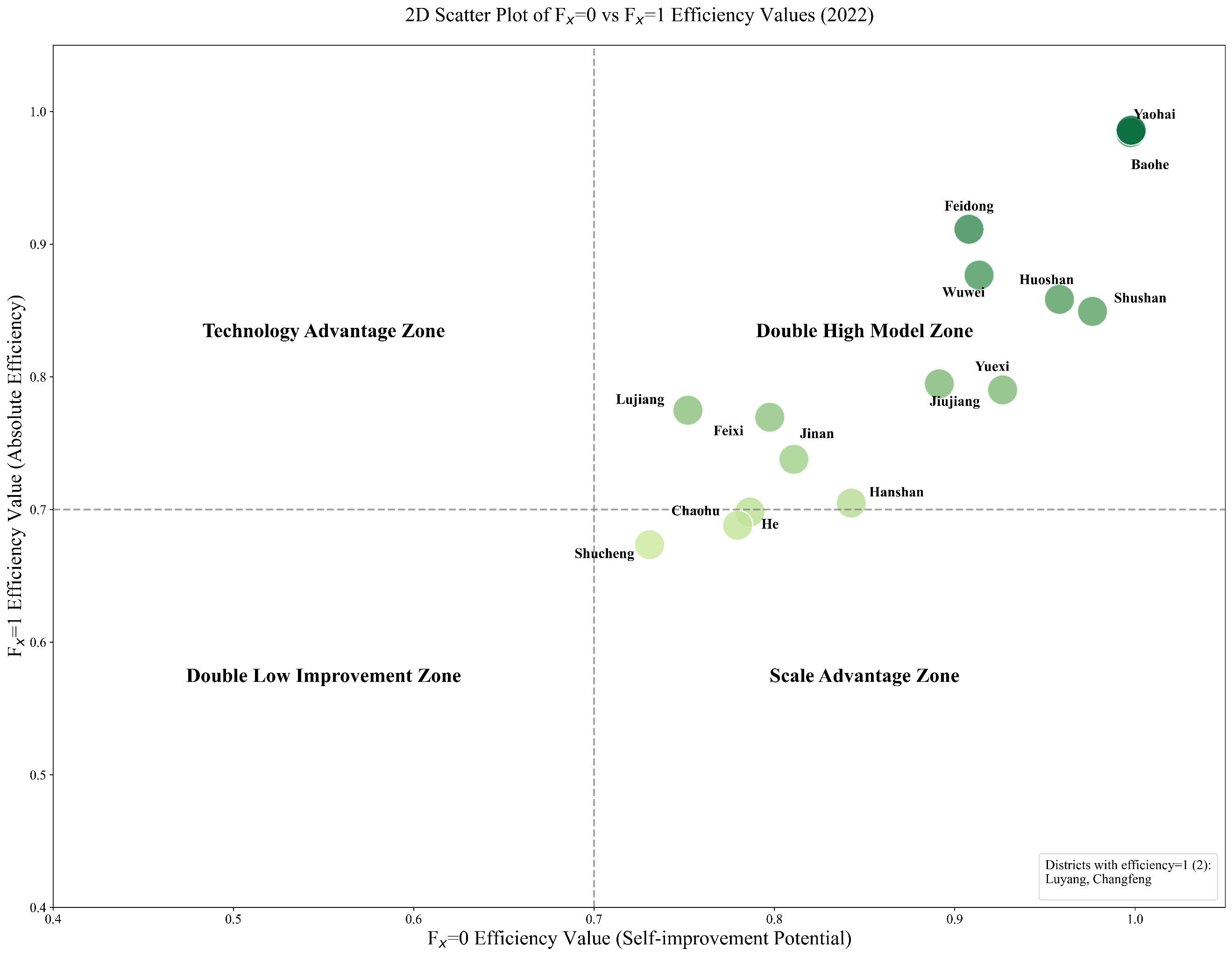


**Figure 7 2022 Efficiency Value Two-Dimensional Scatter Plot**
